# High-resolution accurate mass- mass spectrometry based- untargeted metabolomics: Reproducibility and detection power across data-dependent acquisition, data-independent acquisition, and AcquireX

**DOI:** 10.1016/j.csbj.2025.05.046

**Published:** 2025-05-30

**Authors:** Hanane El Boudlali, Laura Lehmicke, Uta Ceglarek

**Affiliations:** Clinical Mass Spectrometry Section, Institute of Laboratory Medicine, Clinical Chemistry and Molecular Diagnostics, University of Leipzig Medical Center, Liebig Street 27, Leipzig D-04103, Germany

**Keywords:** AcquireX, DDA, DIA, detection power, reproducibility

## Abstract

Untargeted metabolomics aims at the unbiased metabolic profiling and biomarker discovery but requires methods with high sensitivity and reproducibility. Here, we compare three acquisition modes—Data-Dependent Acquisition (DDA), Data-Independent Acquisition (DIA), and AcquireX —to evaluate performance and reproducibility in detecting low-abundance metabolites in a complex matrix. A system suitability test (SST) based on 14 eicosanoid standards was implemented to evaluate the suitability of our instrumental setup prior to conducting untargeted metabolomics analyses and monitor long-term system performance. Bovine liver total Lipid Extract (TLE) was spiked with decreasing levels (10–0.01 ng/mL) of the eicosanoid standard mix (StdMix) to compare the detection power of each mode. Reproducibility was evaluated over three independent measurements, spaced one week apart. Chromatographic separation was performed on a C18-Kinetex Core-Shell column and HRAM-MS/MS data were acquired using an Orbitrap Exploris 480. DIA detected and identified the highest number of metabolic features, (averaging 1036 metabolic features over three measurements), followed by DDA (18 % fewer) and AcquireX (37 % fewer). Moreover, DIA demonstrated superior reproducibility, with a coefficient of variance of 10 % across detected compounds over three measurements, compared to 17 % for DDA and 15 % for AcquireX. DIA further exhibited better compound identification consistency, with 61 % overlap between two days, compared to DDA (43 %) and AcquireX (50 %). DIA reproduced fragmentation spectra patterns with high consistency, contributing to higher reproducibility in compound identification. DIA showed the best detection power for all spiking eicosanoids at 10 and 1 ng/mL in TLE matrix. At low spiking levels, 0.1 and 0.01 ng/mL, a general cut-off was observed for the three acquisition modes. None of this assessed acquisition modes was able to detect and/or identify eicosanoids at physiologically relevant concentrations, explaining their frequent omission in routine untargeted analyses.

## Graphical Abstract

**Figure:**
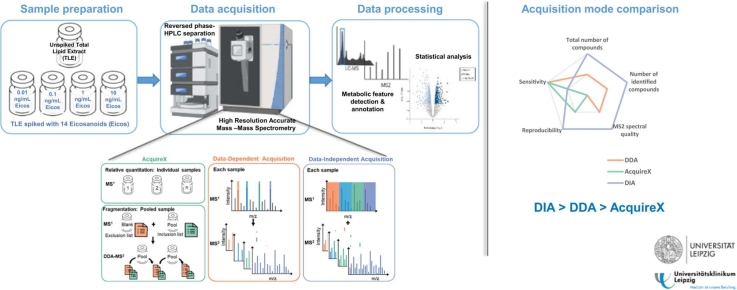

## 1 Introduction

Untargeted metabolomics aims at the unbiased and comprehensive identification of metabolites in organisms, tissues, body fluids, or cells [1], [2]. In the clinical setting, untargeted metabolomics has served, over the last years, as a global molecular profiling strategy in biomarker and metabolic pathway research [3], [4], [5], [6] and in the study of diseases [4], [7], [8]. Identification of candidate biomarkers by untargeted metabolomics remains challenging due to a high variability in chemical structures, isomeric/isobaric compounds and the broad dynamic concentration range of metabolites in biological samples [9]. The combination of complementary and orthogonal techniques has emerged as the possible path to an extensive metabolite coverage [10]. Chromatographic separation and subsequent high-resolution accurate mass- tandem mass spectrometric (HRAM-MS/MS) detection represent the best tools to resolve some of the complexity of biological samples and extend the comprehensiveness of its analysis [11], with high-performance liquid chromatography (HPLC)- HRAM-MS/MS variant being the most commonly used for metabolite profiling [12]. HPLC provides versatile separation capabilities across a broad range of metabolite polarities, depending on the choice of stationary phase and chromatographic mode. The high resolution of HRAM-mass spectrometers, e.g. Fourier transform- ion cyclotron resonance (FT-ICR) MS, Orbitrap MS, and time-of-flight (TOF)- MS, enables the separation of isobaric metabolites and, along with instrument calibration, the measurement of near exact masses to extract metabolic features with mass accuracies below 5 ppm [12]. On state-of-the-art platforms, mass accuracies below 1 ppm are achieved, which reduces the number of possible molecular formulae for the detected mass-to-charge ratios (*m/z*). At high resolution, these possible molecular formulae are further reduced by heuristic filtering based on fine isotope structure [13]. Tandem MS (also MS/MS or MS^2^), in hyphenated instruments such as the Quadrupole-Orbitrap or Quadrupole-TOF, generates fragmentation spectra, i.e. fragments the precursor ions into smaller product ions, necessary for identifying the small molecules [14]. These MS^2^ spectra can be matched with compounds in spectral libraries to achieve the 2^nd^ highest confidence level in metabolite identification in an untargeted analysis. The highest level of identification is achieved by validating at least two independent and orthogonal data points (e.g., retention time, accurate mass, and/or MS^2^ spectra) against authentic standards measured on the same instrument and under identical conditions [15], [16]. Building on this identification strategy, the generation of MS^2^ spectra becomes a crucial step in untargeted workflows.

MS^2^ data can be acquired using either data-dependent acquisition (DDA) or data-independent acquisition (DIA), each affecting the metabolome coverage differently [14]. In DDA mode, the mass spectrometer cycles between a full scan and a set of data-dependent MS^2^ (ddMS^2^) scans generated by the fragmentation of a subset of precursor ions selected from the full-MS scan spectrum [17]. This selection is based on user-defined criteria, such as an intensity threshold, a top-N rule—where only the N most intense precursor ions are selected for fragmentation—and the charge state of the detected precursors [18]. DDA generates MS^2^ spectra of the selected ions in narrow isolation windows; typically 1–1.5 *m/z*. Widening and predefining the isolation windows result in the DIA mode [18]. In such experiments, the acquisition of MS^2^ spectra does not depend on the full-MS scan, so the latter can theoretically be omitted. However, in untargeted metabolomics, full scans are typically retained to support robust feature detection, alignment, and relative quantitation. Different DIA techniques are available depending on the manufacturer and differing in isolation window widths and multiplexing [12]. Variable DIA (*v*DIA), for instance, is accessible on the Orbitrap and it consists of user-defined isolation windows of variable widths. Another conventional method for acquiring MS² spectra separates the full scan from MS^2^-spectral acquisition and therefore relative quantitation from structure elucidation [19]. In this approach, generally, study samples are analyzed in full scan only and pooled samples are analyzed in DDA or DIA modes to acquire fragmentation spectra. Recently, Thermo Fisher Scientific has incorporated this approach into the fully automated data acquisition software, AcquireX (Waltham, MA, USA), especially designed for small molecule analysis. AcquireX’s deep scan workflow creates an exclusion list from blank samples to exclude background ions from fragmentation. A full scan is performed on a pooled sample to create an inclusion list with all ions detected. A DDA- based method is then used to fragment ions in the pooled sample iteratively, i.e. inject the pooled sample n times (n ≥ 2). Based on the target masses from the inclusion list, inclusion and exclusion lists are dynamically updated to prioritize new ions in subsequent replicate analyses. Full- MS scan is performed on each study sample to capture accurate *m/z* and relative abundances for all detected features.

A comprehensive study meticulously evaluated the strengths and weaknesses of the full-scan, DDA and DIA modes usually employed in untargeted metabolomics [19]. While DIA provides broader coverage of metabolic features and higher MS^2^ spectra quantity than DDA, the MS^2^ spectral quality is higher for DDA [19]. For DIA, coeluting ions within one *m/z* window are fragmented together and their deconvolution requires sophisticated informatics tools. Extending these findings, another study assessed AcquireX and its processing software Compound Discoverer (CD) and reported that AcquireX increased the number of potentially identifiable compounds by 50 % through six iterations of MS^2^ acquisition [20]. A shortcoming of this approach is that generating exclusion and inclusion lists, as well as the high number of iterations during the analysis of pooled samples for exhaustive MS^2^ acquisition, may require additional instrument time and sample volume. One key consideration when working with AcquireX™ is its dependency on precise and reproducible chromatographic separation. Inclusion and exclusion lists are built on both precursor *m/z* and retention time acquired from two different samples, which are separately analyzed and compared. Therefore, poor chromatographic reproducibility, reflected in retention time shifts (e.g. ≥1 %), can widen the targeting windows for a given precursor *m/z* ion and reduce selectivity of exclusions and inclusions and the accuracy of their updates. Consequently, closely eluting isomers could be missed. Similarly, insufficient mass resolution or instable mass accuracy may hinder the distinction of co-eluting isobars with small mass differences (e.g. below 10 ppm), affecting the quality of MS² acquisition [21]. Another significant limitation of AcquireX™ is its exclusive availability to Thermo Scientific Orbitrap users and its cost barrier.

Despite the key findings provided by these and other studies, a critical research gap remains. Whereas it is known that untargeted metabolomics approaches are shown to identify high-to medium abundance metabolites, the identification power of these methods for low-abundance metabolites has not yet been systematically investigated. One class of compounds particularly affected by these limitations are lipid mediators, derived from polyunsaturated fatty acids (PUFAs), which play key roles in regulating cell proliferation, tissue repair, coagulation, and immune responses [21]. Eicosanoids, for instance, are formed from PUFAs through cyclooxygenase (COX), lipoxygenase (LOX), and cytochrome P450 (CYP) pathways [22], and play a critical role in inflammation and innate immune responses [23]. However, their low endogenous concentrations in human plasma—ranging from low pg/mL for prostaglandins and leukotrienes up to low ng/mL for hydroxyeicosatetraenoic acids (HETEs) and hydroxyoctadecadienoic acids (HODEs)—make their detection particularly challenging [9], [24], [25]. In contrast to highly sensitive targeted LC-MS/MS approaches previously reviewed [22], untargeted metabolomics often overlooks these low-abundance metabolites due to the inherent limitations in method sensitivity and selectivity and to matrix effects. Furthermore, the presence of multiple isomeric structures within this class underscores the critical need for methods capable of accurate structural elucidation with high day-to-day reproducibility.

To our knowledge, no study to date has directly compared DDA, DIA, and AcquireX within a single, comprehensive analysis. Moreover, there has been no systematic evaluation of each method’s reproducibility and detection power across repeated measurements over days or weeks, particularly for low-abundance metabolites. To address this gap, we assessed the overall detectability and reproducibility of each acquisition mode using a diluted total lipid extract (TLE) from bovine liver, selected to represent a complex biological matrix. Beyond this general assessment, our study aimed to answer two specific questions:

1. Which acquisition mode most reliably identifies low-abundance metabolites with similar chemical structures?
2. Which mode is best suited to distinguish low-abundance features between two biological states, such as diseased versus healthy conditions?

To answer the first question, we focused on 14 pathophysiologically relevant eicosanoids. This class of analytes spans a broad polarity range (low to medium) and features structurally similar compounds. As they are well-characterized in our laboratory [9], these eicosanoids provided a controlled framework for evaluating both detection sensitivity and identification accuracy using known standards. To address the second question, we spiked four different concentrations of the eicosanoid standards mix into the same TLE matrix and compared the spiked samples to unspiked controls. This design enabled us to assess each method’s ability to detect and identify low-abundance features in a complex biological context, simulating real-world biomarker discovery scenarios.

In parallel, we developed a system suitability testing (SST) platform based on the same eicosanoid standards to verify instrument performance before untargeted metabolomics analyses. This SST platform ensures measurement reliability and reproducibility, while also enabling a fair comparison among the three acquisition modes. TLE samples were only injected once SST results met predefined criteria for mass accuracy, peak area reproducibility, and retention time stability.

## 2 Materials and methods

### 2.1 Chemicals and solvents

UHPLC-MS grade acetonitrile (ACN), methanol (MeOH), isopropyl alcohol (IPA), and 99 %; ULC/MS CC/SFC grade formic acid (FA) were purchased from Biosolve (Valkenswaard, The Netherlands) and n-hexane from Supelco, Merck KGaG (Darmstadt, Germany). HPLC–MS grade water (H_2_O) (GenPure) was produced in house with a Barnstead Nanopure system (Thermo Fisher Scientific, Waltham, MA, USA). 2,6-di-tert butyl-4-methylphenol (BHT) was purchased from Sigma-Aldrich (St. Lois, MO, USA). The TLE of bovine liver was obtained from Avanti Polar Lipids Inc. (supplied by Merck KGaA, Darmstadt, Germany) and the eicosanoid standards from Cayman Chemical (Michigan, USA). The list of the 14 standards and their properties is available in the supporting information (SI), table S1. The Pierce™ FlexMix™ Calibration Solution was purchased from Thermo Fisher Scientific GmBH (Dreieich, Germany).

### 2.2 Sample preparation

The standard mixture (StdMix) consists of the 14 eicosanoid standards with a concentration of 50 ng/ mL each. The StdMix was used as an SST-standard to monitor the LC-MS suitability prior, during and after untargeted measurement. The bovine liver total lipid extract is obtained as solution in chloroform with 25 mg/ mL of bovine liver total lipids. It was diluted in 1:1 v/v H_2_O:MeOH by a factor of 100 to give the unspiked TLE sample. To assess the sensitivity of the different acquisition methods, TLE was spiked with the 14 eicosanoid standards at four concentrations; 10, 1, 0.1, and 0.01 ng/mL, to give the spiked TLE at different levels. Solvent blanks constituted of 1:1 v/v H_2_O:MeOH. BHT was added to all samples at a concentration of 50 µg/mL to prevent oxidation of the eicosanoid standards. All samples were prepared and aliquoted on the same day and they are all only thawed once prior to the measurements.

### 2.3 Instrumental method

Orbitrap Exploris 480 high-resolution mass spectrometer (HRMS) was equipped with an OptaMax Next Generation source in heated electrospray ionization (H-ESI) mode and Dionex Ultimate 3000 RS UHPLC+ focused from Thermo Fisher Scientific GmBH (Dreieich, Germany). Prior to the measurements, the instrument was calibrated following the manufacturer’s recommendation, using the FlexMix™ calibration solution.

#### 2.3.1 Liquid chromatography

The LC separation was optimized based on an established chromatographic separation method for eicosanoids using a Kinetex® Core-Shell reversed-phase column (C18, 100 ×2.1 mm, 2.6 µm, 100 Å) from Phenomenex (Torrance, CA, USA) [9]. The mobile phase A consisted of H_2_O/ ACN 95/5 v/v with 0.05 % FA and mobile phase B was ACN/IPA 50/50 v/v with 0.05 % FA. The gradient was set as follows: 0 min 100 % A, 35.9 min 0 % A, 40.9 min 0 % A, and 41 min 100 % A. The post-gradient equilibration time was 7.5 min at 100 % A. The flow rate was 0.5 mL/min and the injection volume 25 µL. The column oven and auto-sampler temperatures were kept at 50 °C and 10 °C, respectively.

#### 2.3.2 HRAM-MS acquisition programs

This study focused on comparing different data acquisition methods in the negative-ion mode only, as it covers all compounds of interest. Ion source parameters and MS settings were the same for all methods. Nitrogen was used as sheath gas, auxiliary gas, and sweep gas with flow rates 50, 10, and 1 arbitrary units (a. u.), respectively. The following parameters were set; spray voltage, 2.5 kV (negative); S-Lens RF level, 40 %; vaporizer temperature, 350°C; and ion transfer tube temperature, 325°C. The MS acquisition time was 0–45 minutes without mass lock.

**Full-scan-** The SSTs were measured in full scan (MS^1^) at a resolution of 120,000 at *m/z* 200 with a scan rate of 3 Hz. The scan range was *m/z* 180–1200 with one microscan. Standard AGC target was used and the maximum injection time was set to auto-mode.

**MS**^**2**^**- scans-** The different acquisition modes included a full-scan, described above, followed by either a data-dependent MS^2^ scan (ddMS^2^), as for DDA and AcquireX™, or a DIA- MS^2^ scan. For comparability, the instrument parameters for all MS^2^ -scans were the same for all acquisition modes, namely resolution 30,000 at *m/z* 200 and scan rate 12 Hz; mass range, *m/z* 180–1200; normalized stepped collision energies, 30, 50 %; RF-lens, 50 %; and microscans, 1. AGC target was set to standard and maximum injection time was in auto-mode.

**DDA-** following a full scan, a ddMS^2^ was performed using the following filters: intensity threshold at 1.0e3; dynamic exclusion after 1 detection for 10 s; mass tolerance; 5 ppm, with isotope exclusion. The cycle time was set to 0.6 s between master scans and the microscan number was 1.

**AcquireX**^**TM**^ Data Acquisition software (Thermo Fisher Scientific, Waltham, MA, USA**) -** Deep scan workflow was applied to ensure in-depth identification of metabolites. Two types of instrument methods were used: full scan (see parameters above) and a ddMS^2^ with the same parameters as DDA but with two additional filters: an inclusion list and an exclusion list both created and updated by AcquireX™ software on the fly. Using a full-scan only, one solvent blank was injected to create the exclusion list and one ID- TLE sample at the lowest spiking concentration of eicosanoid standards (0.01 ng/mL) was injected once to create an inclusion list. Four iterative injection of the ID-TLE sample were performed to produce exhaustive MS^2^ data by automatically updating the inclusion and exclusion lists. The mass tolerance for the mass targets was 10 ppm in both lists. Study samples, i.e. unspiked and all spiked TLE samples, are then injected in full scan only to perform relative quantitation.

***v*****DIA-** the acquisition occurred in two independent experiments; the first being the full-scan and the second the DIA with variable isolation window width. For precursor range 180–700, the *m/z* window was 20 amu and for the range 700–1120 it was 50 amu. The exact DIA windows are listed in the SI table S2. The MS cycles between both experiments during the chromatographic run.

#### 2.3.3 Experimental design

To compare the detection power and reproducibility of the three acquisition modes in untargeted metabolomics, TLE both unspiked and spiked with decreasing concentrations of eicosanoid standards, were analyzed using each method. TLE samples were aliquots of the same TLE standard, subjected to identical preparation steps and the same number of freeze/thaw cycles. Randomization, blocking, and replication were employed to minimize batch effects, instrumental drift, or potential carry-over effect. The study was designed such that each week, a different acquisition mode was initiated, and within each mode’s sequence, the order of sample analysis was totally randomized to prevent systematic bias. This blocking and randomization strategy ensured that no acquisition mode or sample order was repeated in a consistent pattern across runs. Each complete cycle of measurements spanned three days, during which SSTs, i.e. StdMix, samples were measured to monitor instrument performance and maintain data integrity.

#### 2.3.4 Data processing

All data processing was carried-out on a computer with an Intel i5–8500 CPU @ 3.00 GHz with six cores and 16 GB memory, Windows 10, 64-bit operation system, and six logical processors. For the SST, the analysis was conducted in a targeted manner using Freestyle™ 1.8 SP2 QF1 (version 1.8.65.0), the exact chromatographic ranges are shown in the SI, table S3. Compound Discoverer version 3.3 (CD3.3) service pack 1 was used for untargeted analysis of TLE samples. For comparability purposes, the same workflow was used for all three methods. mzCloud is the only MS^2^ database search that supports DIA analysis in CD3.3, therefore it was the only one used for MS^2^-spectral querying. Detailed description of the workflow is presented in the SI, figure S1 to S3 and table S4. The raw data are publicly available in Metabolights data repository under accession number MTBLS12126.

#### 2.3.5 Acquisition mode comparison

The three acquisition modes were compared according to several metrics. The first two metrics, the number of detected metabolic features and their identification performance, were evaluated with unspiked TLE samples. Reproducibility in detection and identification was assessed over three independent measurements one week apart, also using unspiked TLE samples. Identification quality and accuracy were further assessed using spiked TLE samples also analyzed in an untargeted manner. This was achieved by manually verifying the annotation of the spiking eicosanoid standards using *m/z* and RT data obtained from our SST, which included the same set of analytes. MS^2^ spectral reproducibility were also evaluated for these eicosanoid standards. For AcquireX, the identification samples (i.e., samples used to generate MS^2^ spectra) were ID- TLE spiked at the lowest concentration (0.01 ng/mL), aiming to test AcquireX's capability to generate MS^2^ spectra for trace compounds. The final metric, sensitivity or detection power of low-abundance metabolites as biomarker candidates, was assessed by comparing spiked to unspiked TLE samples. Upregulated analytes were defined as metabolic features with adjusted p-values lower than 0.05 and fold-change (FC) greater than 2. Additional details on the differential analysis are provided in table S4 of the Supporting Information. The detection and identification of the standards were manually verified based on their known RT and *m/z* values. The results shown in the following are after background removal. Compounds are marked as background if present in the first and last blank samples of each sequence according to the parameters shown in figure S1, “Mark Background Compounds” node.

## 3 Results

### 3.1 System suitability tests

To assess system suitability, StdMix samples containing 14 eicosanoid standards covering a broad polarity range were analyzed a priori throughout the measurement period. Only when the mass accuracy was within the + /- 5 ppm mass tolerance limit, untargeted measurements were conducted. Coefficient of variation of maximally 20 % for peak areas and 2 % of RTs were tolerated. Otherwise the mass calibration was repeated and instrumental maintenance was performed.

**Fig. 1:**
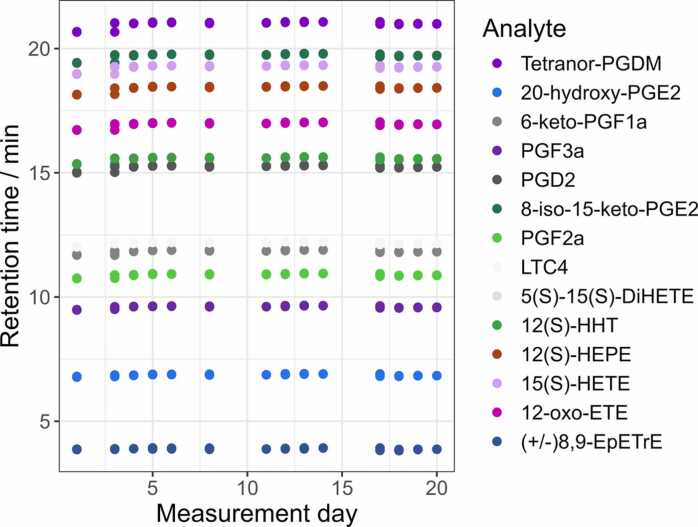
Retention times (min) of 14 eicosanoid standards measured in StdMix samples over 21 days. The standards, present at 50 ng/mL each with 50 µg/mL BHT in 1:1H₂O:MeOH (v/v), were analyzed in quadruplicate on each day. Chromatographic separation was performed using a Kinetex® C18 (100 × 2.1 mm, 2.6 µm) column with a flow rate of 0.5 mL/min. The mobile phases consisted of H₂O/ACN (95:5, v/v, 0.05 % FA) as phase A and ACN/IPA (50:50, v/v, 0.05 % FA) as phase B. The gradient was 0 min 100 % A, 35.9 min 0 % A, 40.9 min 0 % A, and 41 min 100 % A, followed by 7.5 min re-equilibration. The column and autosampler were maintained at 50 °C and 10 °C, respectively.

To ensure comparable analytical conditions for the three acquisition modes and that any variation is due to the method and not instrumental drift, the stability of the instrumental setup was monitored. On each measurement day, two StdMix injections were performed prior to, one in the middle of a sequence, and one afterwards. The RTs, fig. 1, show stability throughout the measurement period with a CV of 0–1 % (table 1) applying the optimized chromatographic conditions. Table 1 displays the peak areas stability for the monitored eicosanoids with CVs between 5 % and 14 %. The absolute mass accuracies are within 5 ppm window defined in the untargeted workflow. The mean values of RTs and peak areas are listed in table S5, in the SI.

**Table 1:** Retention time (RT) and peak area variability, and mass accuracy of 14 eicosanoid standards included in the SST mix (50 ng/mL each + 50 µg/mL BHT in 1:1H₂O:MeOH, v/v), measured in full-scan (MS^1^) mode over 21 days (quadruplicate injections per day). RT and peak area variability are reported as coefficients of variation (CV, %), and mass accuracy as mean ± 2 SD (ppm). All measurements were performed under consistent chromatographic and MS conditions in negative-ion mode.

Analyte	RT	Peak Area	Mass accuracy	
	CV/%	CV/%	Mean - 2 SD / ppm	Mean + 2 SD / ppm
Tetranor-PGDM	1	6	3.66	−0.48
20-hydroxy-PGE2	1	5	3.89	−0.71
6-keto-PGF1α	0	7	4.01	−0.66
PGF3α	0	7	3.81	−0.59
PGD2	0	7	3.81	−0.59
8-iso−15-keto- PGE2	0	5	3.51	−0.45
PGF2α	0	7	4.17	−0.63
LTC_4_	0	8	3.89	−0.61
5(S),15(S)-DiHETE	0	7	3.60	−0.55
12(S)-HHT	0	12	3.53	−0.54
12(S)-HEPE	0	7	3.56	−0.47
15(S)-HETE	0	8	3.53	−0.54
12-oxo-ETE	0	8	3.53	−0.56
(+/-)8,9-EpETrE	0	14	3.49	−0.46

### 3.2 Acquisition modes comparison

#### 3.2.1 Detection and identification performance

To compare the three acquisition —AcquireX, DDA, and DIA—we first analyzed a 100-fold dilution of the commercially available bovine liver TLE without spiking. All measurements were performed in negative-ion mode using parameters specific to each acquisition mode as detailed above. CD3.3 was selected for feature extraction, alignment, and metabolite annotation and processing parameters were optimized to achieve the best performance of metabolic feature extraction. This initial comparison was designed to assess the overall detection and identification capabilities of each acquisition strategy. Feature counts were categorized into (i) detected features after background subtraction (see details in figure S2, SI); (ii) MS^1^-annotated features with *m/z* matches to at least one compound in mass lists or ChemSpider; and (iii) MS^2^-matched features with spectral similarity to reference fragmentation spectra in mzCloud. Further details on the MS^1^ and MS^2^- matching is presented in figure S3, in SI.

**Fig. 2:**
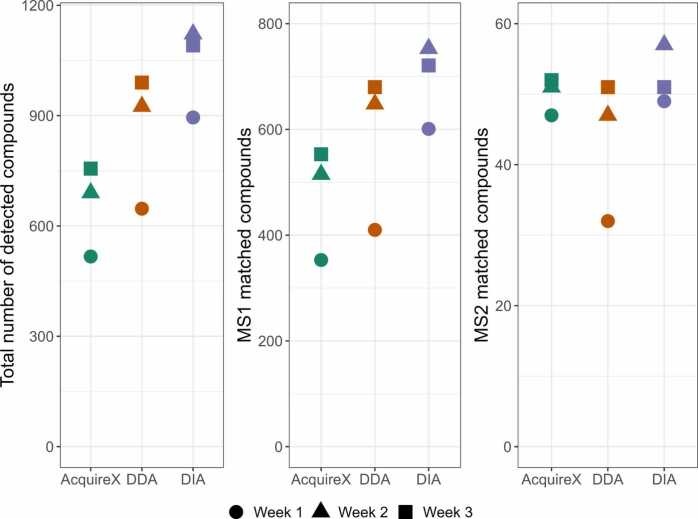
Comparison of the number of detected features (left), MS^1^-annotated features (middle; based on mass list and ChemSpider), and MS^2^-matched features (right; based on mzCloud) across three acquisition modes: AcquireX, DDA, and DIA. Analyses were performed in negative-ion mode on unspiked total lipid extract (TLE) samples. Each data point represents the mean of three replicate injections per mode per week. Values shown are after background subtraction. The calculated MS^2^-based identification rate relative to total detected features is 7.6 % for AcquireX and 5 % for DDA and DIA.

As shown in fig. 2 and summarized in table 2, DIA outperformed DDA and AcquireX in the number of detected and MS^1^-matched features. Over the three-week measurement period, the total number of detected and identified features generally increased, except for DIA, which peaked in week 2. Temporal variation was particularly pronounced between weeks 1 and 2 for detected and MS^1^-annotated features, but no corresponding trend was observed in SST peak areas (figure S4, SI). Comparing the spiking standards in TLE matrix, a slight increase in standard intensities was observed across acquisition modes from week 1 to weeks 2 and 3 (tables S6–S8, SI). For MS^2^ matches, absolute numbers remained below the typical 10 – 20 % yield observed in untargeted metabolomics, but AcquireX outperformed DDA and DIA with an MS^2^ identification rate of 7.6 % relative to total detected features across the three measurements, compared to 5 % for both DDA and DIA. When evaluating reproducibility across weeks using the same sample, AcquireX exhibited the highest variability in feature counts, followed by DDA, with DIA showing the most consistent performance.

**Table 2:** Comparison of acquisition modes (AcquireX, DDA, and DIA) based on total detected features and identification performance. Values represent the mean and coefficient of variation (CV, %) over three weekly measurements as presented in fig. 2 (each weekly measurement is derived from triplicate injections of unspiked TLE samples). MS^1^-based identifications were performed using mass list and ChemSpider, while MS^2^-based identifications were matched using mzCloud. All values are after background feature removal and were acquired in negative-ion mode.

		Total detected	MS^1^ -match	MS^2^ -match
AcquireX	Mean	654	474	50
	CV / %	15	18	4
DDA	Mean	854	579	43
	CV / %	17	21	19
DIA	Mean	1036	692	52
	CV / %	10	9	6

**Fig. 3:**
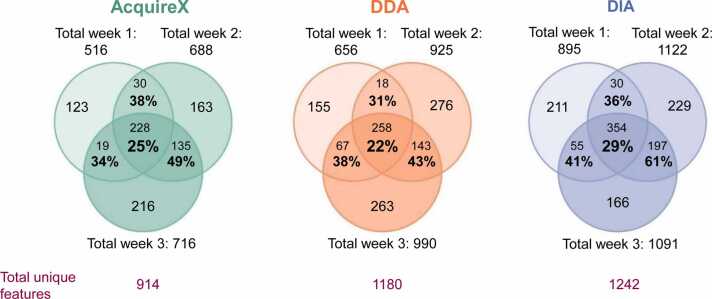
Overlapped MS^1^ and MS^2^ features in TLE samples across three independent measurements acquired using AcquireX, DDA, and DIA modes. The overlap between all three weeks was calculated as the proportion of features shared across all datasets relative to the total number of unique (non-redundant) features detected in the three measurements. Similarly, pairwise overlaps between two weeks were calculated as the proportion of features shared between the respective datasets relative to the total number of unique features observed in those two weeks.

After comparing the feature counts and their variability across weekly measurements, we next examined whether the same compounds were consistently identified each week. Specifically, we asked whether similar numbers of features across weeks reflected reproducible identification of the same metabolites, or if different subsets were being annotated in each run. To evaluate this, annotated features from the three independent weekly measurements were compared to assess overlap in feature identity. In the Venn diagrams in fig. 3, the overlaps represent the proportion of shared features relative to the total number of non-redundant features identified with at least an MS^1^ match. While table 2 and fig. 2 report total feature counts including redundant identifications, the values in fig. 3 focus exclusively on unique, non-redundant features identified by at least an MS^1^-match. The overlap between two or three weeks is calculated as the proportion of shared metabolic features relative to the total number of non-redundant features identified in both or the three weeks combined. AcquireX showed overlaps of 38 %, 34 %, and 49 % between weeks 1 & 2, 1 & 3, and 2 & 3, respectively. DDA exhibited overlaps of 31 %, 38 %, and 43 % for the same comparisons. DIA yielded the highest reproducibility, with overlaps of 36 %, 46 %, and 61 %.

#### 3.2.2 Accuracy and reproducibility of identification

**Fig. 4:**
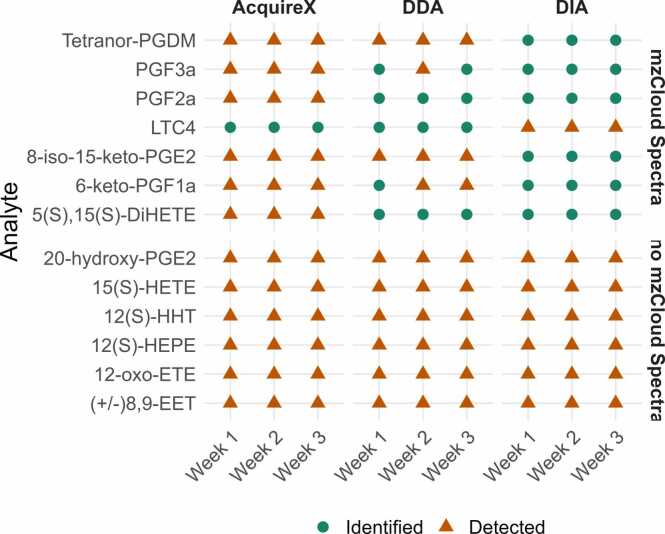
Identification accuracy and reproducibility of eicosanoid spiking standards analyzed using AcquireX, DDA, and DIA across three independent weekly measurements. The figure includes three panels, one per acquisition mode, with the 13 eicosanoid standards plotted on the y-axis and the weekly measurements on the x-axis. MS¹ and MS² data were acquired from TLE samples spiked at 10 ng/mL for DDA and DIA. For AcquireX, MS¹ data were acquired at 10 ng/mL spiking, whereas ID-TLE samples used for MS² acquisition were spiked at 0.01 ng/mL. Data were processed using an untargeted workflow, with feature annotation based on publicly available libraries (MS¹: ChemSpider and mass lists; MS²: mzCloud; see Figs. S2 and S3 in SI). Detection was defined as the presence of a corresponding feature with a mass error ≤ 5 ppm and consistent retention time. The 14 eicosanoid standards were grouped into two panels: the top panel includes standards with available reference spectra in mzCloud used for MS²-based matching, while the bottom panel contains those without mzCloud reference spectra. Correct annotations are indicated with green circles and incorrect annotations with orange triangles.

Beyond the assessment of overall sensitivity and reproducibility using unspiked TLE samples, we next aimed to address a more specific question: which acquisition mode can reliably identify low-abundance metabolites with similar structures? To evaluate identification accuracy and reproducibility, we analyzed TLE samples spiked with eicosanoid standards. Details on the spiking standards, including molecular formulae, purity, and physicochemical properties, are provided in figure S1 in the SI. To increase the level of complexity, coeluting isomeric compounds (e.g. PGD₂ & PGF₃α) were included, along with metabolites for which reference spectra are available in the mzCloud database (in negative mode, across different activation energies), and others for which no reference spectra exist. Fig. 4 presents results from the comparison of the three acquisition modes across weekly measurements, using the highest spiking level, i.e. 10 ng/mL. At this spiking level, all standards were detectable allowing us to assess the identification accuracy and reproducibility. For AcquireX, MS² spectra were generated, separately from MS^1^ scan, at a spiking concentration of 0.01 ng/mL. The use of this lower concentration for AcquireX was intentional, as Thermo Scientific promotes it as a sensitive profiling workflow capable of detecting trace metabolites—especially in deep scan mode—and we aimed to evaluate this aspect. While MS² spectra were successfully acquired for all spiked standards at 10 ng/mL using DDA and DIA, most standards did not yield MS² spectra with AcquireX at the lower concentration. Several reasons may explain this outcome: the signal intensities at 0.01 ng/mL may have fallen below the MS² triggering threshold (10 ³), more iterations may be needed for ID analysis in AcquireX, or spectra could have been filtered out during software processing. Fig. 4 categorizes the standards into two groups: the top block includes compounds with MSⁿ reference spectra available in mzCloud, while the bottom block shows those without such entries.

For standards without mzCloud reference spectra, annotation was incorrect across all acquisition modes and weekly measurements. However, two scenarios were identified. In **Case A**, no mzCloud reference spectra existed for any given precursor ion with *m/z* within the 5 ppm mass tolerance window (e.g. 15(S)-HETE and (+/-)8,9-EET). Here, annotations depended solely on reproducible MS¹ -based matching and thus were incorrect but consistent across all methods. In **Case B**, mzCloud reference spectra existed for unrelated entries with precursor ions that fall within the mass tolerance for spectral matching. In this case, the reproducibility of annotation varied depending on the acquisition mode. DIA consistently acquired identical MS² spectra, resulting in consistent but incorrect MS^2^-based annotations across weekly measurements. Conversely, DDA annotations were incorrect and inconsistent because MS² spectra exhibited spectral differences between weeks. Table S9 in SI shows the case of 12(S)-HEPE: DIA consistently annotated it as (+)-18-HEPE (confidence ∼50 %), while for DDA we observed MS² spectra with differing fragmentation pattern. This could be due to a coeluting isomer or a mismatching of MS^1^ and MS^2^ features. AcquireX did not generate any MS² spectra for this standard at the level used for MS^2^ acquisition.

For standards with available mzCloud reference spectra, AcquireX consistently provided incorrect annotations for most eicosanoids, DDA annotations were inconsistent across measurements, and DIA correctly and consistently annotated most of these compounds. Three annotation behaviors were observed and are illustrated with examples in the following. **Case C** included standards for which MS² spectra were consistently acquired, yet annotations were based solely on MS¹ -level matching. A representative example is LTC4, for which no MS²-based annotation was obtained with DDA, despite the presence of a reference spectrum. Inspection of the MS² spectra generated by DDA for LTC4 (table S10, SI) revealed the absence of the precursor ion, possibly due to suboptimal fragmentation timing (e.g., fragmentation occurring away from the peak apex) or incorrect precursor-product ion pairing during data processing. This resulted in annotations relying exclusively on MS¹ -level matching. In C**ase D**, similar to **Case B**, spectra with differing fragmentation pattern were observed for DDA and AcquireX, leading to inconsistent identification. One illustrative case—6-keto-PGF1α—is shown in table S11. This compound yielded different fragmentation patterns with DDA, hence different annotations across measurements. AcquireX did not generate any MS² spectra for this standard at the level used for MS^2^ acquisition. However, another example is that of 8-iso-15-keto-PGE2, for which AcquireX produced different fragmentation pattern and led to different annotations (see table S12). **Case E** includes an instance where MS^2^ is acquired on one week but absent on the other two by AcquireX leading to different annotations. Table S13 illustrates the example of 5(S),15(S)-DiHETE. At the spiking level of 0.01 ng/mL used for MS^2^ spectral acquisition, the intensity of precursor ions does not allow for triggering of MS² spectra by AcquireX even using the deep scan mode. Therefore, AcquireX did not perform well at the tested concentration, this physiological spiking level was chosen specifically to assess its performance in deep detection and MS² profiling. This was observed for DDA as well at lower spiking level. DIA, in contrast, acquired MS² spectra from predefined windows reproducibly, providing consistent annotations even when matching incorrectly to structural isomers (e.g. LTC_4_ as 11-trans-LTC_4_).

In our experimental design, coeluting isomeric compounds, PGD2 and PGF3α, were included to evaluate annotation accuracy. According to mzCloud and expected fragmentation patterns, PGF3α should yield characteristic fragments at *m/z* 333.2070, 307.1914, 253.1962, 245.1911, 235.1340, 193.1234, and 171.1026, while PGD2 is expected to generate fragments at *m/z* 333.2070, 315.1966, 271.2068, and 233.1183. Upon untargeted analysis, these two isomers were consistently annotated as PGF₃α. Examining the acquired MS² spectra for DDA and DIA (table S14), we observed a prominent parent ion peak at *m/z* 351 along with a mixed fragmentation pattern. More intense fragment ions (highlighted with orange circles) matched the predicted fragments for PGF3α (307, 245, 193, and 171), whereas lower-intensity fragments (highlighted with green circles) corresponded to those expected for PGD2 (315, 271, and 233). AcquireX did not acquire fragmentation spectra at the spiking level used. Additionally, within replicate DDA spectra, variability in the observed fragmentation patterns was noticed, possibly due to differences in ion-sampling times during acquisition.

Regardless of the acquisition mode used, we observed issues in molecular ion annotation, where water-adduct or loss ions were sometimes misassigned as separate metabolites or the molecular ion was attributed to the [M-H_2_O-H]^−1^ or other adducts of another metabolite, leading to incorrect identifications; an example of this was PGF2α, illustrated in table S15.

#### 3.2.3 Detection power for low abundance metabolites

**Fig. 5:**
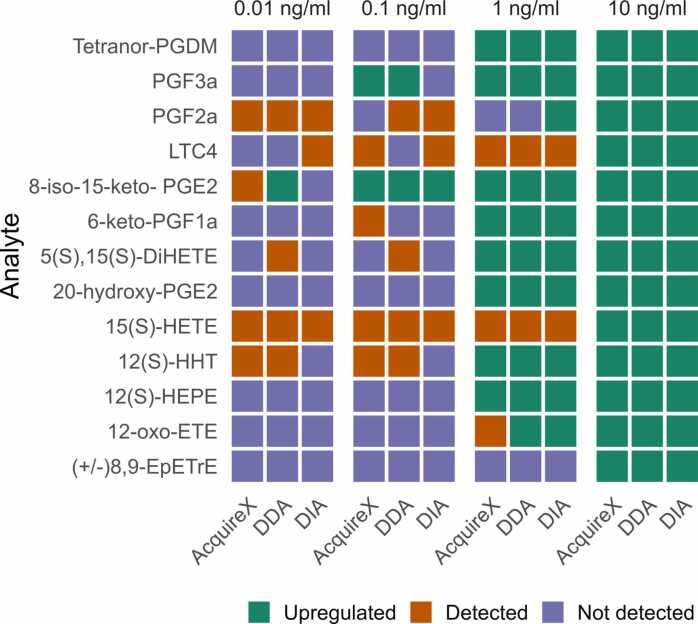
Sensitivity assessment of AcquireX, DDA, and DIA for detecting and differentiating low-abundance eicosanoid standards across four decreasing spiking levels. The figure includes three panels, one per acquisition mode, with the 13 eicosanoid standards plotted on the y-axis and the spiking levels on the x-axis. For each concentration level, a spiked sample was compared to an unspiked TLE sample to mimic a biomarker discovery setup. Detection was defined as the presence of a corresponding feature with a mass error ≤ 5 ppm and consistent retention time. Color coding reflects the outcome for each compound: lilac indicates not detected, orange indicates detected but not statistically significant, and green indicates detection with significant upregulation (p < 0.05 and fold-change ≥ 2). Results are based on data acquired during the second weekly measurement.

To address the second research question—whether different acquisition modes can distinguish low-abundance metabolites between biological states—we designed an experiment using the same TLE matrix spiked with increasing concentrations of eicosanoid standards. This setup was intended to mimic biomarker discovery conditions, where subtle differences in low-abundance compounds may reflect meaningful biological variation. By comparing spiked samples across four concentration levels, i.e. 0.01, 0.1, 1, and10 ng/mL, to unspiked controls, we assessed the sensitivity and consistency of each acquisition strategy in detecting small changes in metabolite abundance within a complex matrix. Fig. 5 summarizes the outcomes for 13 eicosanoids across acquisition modes, highlighting compounds that were not detected (lilac), detected without reaching statistical significance (orange), and detected with significant upregulation (green). In fig. 5, a standard was classified as significantly upregulated if it showed a fold-change of at least 2 and a p-value below 0.05 when comparing the spiked sample to its unspiked control (see details on statistical analysis in table S4 in SI).

At the highest spiking concentration, 10 ng/mL, all three methods detected 13 added eicosanoids as upregulated in the spiked TLE compared to the non-spiked one. PGD2 was not detected separately as it coelutes with PGF3α. At 1 ng/mL, 12 out 13 compounds were detected by DIA compared to 11 for DDA and AcquireX. For DIA, out of these 12, 10 compounds were still considered upregulated in the spiked TLE. At 0.1 ng/mL, acquiring with DDA and AcquireX, 6 features were detected compared to 4 only for DIA and 2 features still considered as upregulated in the spiked compared to only 1 for DIA. At the lowest concentration, i.e. 0.01 ng/mL, the detection power of the spiked components dropped for all methods, but DIA appeared to be mostly affected. AcquireX and DDA still detected 5 standards whereas DIA detected only 3 indicating a compromised sensitivity at lower concentrations. At this concentration, DDA still found 8-iso-15-keto-PGE2 to be upregulated.

## 4 Discussion

In this study, we conducted a comprehensive evaluation of three data acquisition modes—AcquireX, DDA, and DIA—for untargeted metabolomics on an Orbitrap Exploris 480 HPLC-HRAM-MS platform. We compared their overall detectability, reproducibility across repeated measurements, and performance in detecting and identifying low-abundance metabolites. The analysis was carried out using a complex biological matrix (TLE from bovine liver) and 14 eicosanoid standards. These standards were selected from a validated targeted LC-MS/MS method previously developed in our lab, which includes a broader panel of approximately 100 eicosanoids and PUFAs [9]. The selected compounds represent a range of physicochemical properties, including different polarities, molecular weights, and chromatographic retention times. Their known limits of detection (LODs), ranging from 5 to 250 pg/mL on a Triple Quadrupole LC-MS/MS platform (LC-QqQ) [9], provide a useful reference for evaluating the relative sensitivity of untargeted acquisition modes. Although the targeted and untargeted methods differ in instrumentation, acquisition, and data processing, the known LODs from the targeted LC-QqQ method help set realistic expectations for the sensitivity of untargeted acquisition modes and guide interpretation of results—particularly when compounds are not detected. In such cases, absence in untargeted data should not be equated with true absence in the sample, but rather considered in light of the method’s detection limits.

In the first step of the study, we developed and implemented a protocol for monitoring system performance. Stability in retention times (RTs), peak areas, and mass accuracies of the 14 eicosanoid standards—illustrated in fig. 1 and S4 and summarized in table 1 and table S5—demonstrated robust system performance across the three-week measurement period. RTs remained highly stable, with CVs consistently between 0 % and 1 %, despite routine variations in eluent preparation, StdMix handling, and instrument operation. This level of chromatographic reproducibility is critical for untargeted metabolomics, particularly for accurate compound identification and alignment within a batch. It is especially important for AcquireX, where the generation and use of dynamic exclusion/inclusion lists are directly dependent on retention time stability. Mass accuracies remained within the ± 5 ppm tolerance window throughout the measurement period (table 1), helping reduce the identification variability due to mass error.

Reproducibility in peak area measurements is essential for reliable detection and relative quantification, especially in biomarker research. By standardizing a comprehensive LC-HRAM-MS maintenance workflow, we reduced peak area CVs of the 14 SST analytes from initial values of 37–68 % (data not shown) to 5–14 % across three weekly measurements (total of 51 injections over 21 days), with no observable drift nor trend. Routine maintenance included daily flushing of the autosampler and LC system, and rinsing of the HESI probe after each batch to minimize contamination and carryover. Weekly procedures involved mass calibration, cleaning of ion-transfer tube, and monitoring of vacuum pressures, while monthly tasks included deep cleaning of the sweep cone and HESI probe exhaust, along with mass and system calibration. LC pump pressure profiles were regularly checked, particularly after preparing new eluents, to identify potential issues early. As part of the maintenance protocol, deep cleaning of the ion source, bent flatapole, and quadrupole was carried out when deviations in performance were observed.

This quality control approach aligns with current best practices in untargeted metabolomics for system suitability testing and long-term instrument performance monitoring [26]. It also helps minimize batch effects and supports the reliability of downstream statistical interpretation. Importantly, untargeted measurements were only initiated once the predefined SST criteria for RT stability, CVs of 2 %, mass accuracy, + /- 5 ppm, and peak area variability, CVs of 20 %, were met. If acceptance criteria were not reached, maintenance steps such as re-calibration or system cleaning were performed before proceeding with sample acquisition. This aimed to ensure that observed differences between AcquireX, DDA, and DIA primarily reflect differences in acquisition strategy rather than uncontrolled technical variability. We therefore strongly recommend the inclusion of sample independent- SSTs in the daily routine, with clearly defined acceptance criteria, as a means to ensure both system suitability and the effectiveness of ongoing maintenance.

The results presented in fig. 2 and summarized in table 2 compare the detection and identification performance of AcquireX, DDA, and DIA based on the total number of detected features, MS¹ -based annotations, and MS² -based identifications. Across all three acquisition modes, we observed a notable increase in feature counts between week 1 and weeks 2 and 3. However, this pattern was not reflected in the SST metrics, which remained stable throughout the three-week period in terms of retention times, peak areas, and mass accuracies with no observable trends (figure S4, SI). This indicates that while SSTs are effective for confirming that the instrument is operationally stable and ready for analysis, they are not designed to capture more subtle, matrix-dependent variations in signal intensity or detection sensitivity. Consequently, although no major drift or performance degradation was observed, the increasing trend in detected features suggests that additional factors—such as ion source stability, sample matrix effects, or acquisition-specific sensitivities—may contribute to variability across weeks.

Closer inspection of the eicosanoid intensities in spiked TLE samples at 10 ng/mL (figures S6–S8, SI) revealed lower signal intensities during week 1 and increased signals in weeks 2 and 3 across acquisition modes. This fluctuation in instrumental performance—potentially related to factors such as minor shifts in spray stability, gradual source contamination, variation in ion optics transmission, or column conditioning—may partially explain the generally increasing trend in feature counts observed in fig. 2 [26], [27]. Interestingly, DIA showed its highest number of detected and annotated features in week 2 rather than week 3. When reviewing the acquisition order, DIA was measured first in week 1, last in week 2, and second in week 3. If minor sample carryover or cumulative sensitivity effects occurred, particularly in week 2 where DIA followed DDA and AcquireX, this could have contributed to the elevated feature counts despite background subtraction. While we did not observe clear signs of carryover in the SSTs, such mode- and order-dependent effects are known to influence detection sensitivity in untargeted workflows [28]. These findings underscore the limitations of standard SSTs alone and point to the potential benefits of using matrix-matched SSTs or pooled QC samples throughout the sequence. Such controls would offer better resolution of intra- and inter-batch variability. Furthermore, normalization methods such as LOESS or spline regression, widely applied in untargeted metabolomics, correct for drift or batch effects across time, injection order, or measurement blocks, particularly when pooled QC samples are injected regularly [26], [27]. However, these approaches rely on the assumption that QC samples accurately represent the biological matrix, which may not always hold [26].

In our case, a mode-specific QC-based normalization across weeks would have reduced variability within each mode. However, it could have also introduced scaling differences that affect comparability across modes which is central to our study. Moreover, the primary aim of this part of the study was to evaluate the inherent variability of each acquisition mode under standardized conditions. All test samples were prepared in parallel from a single TLE batch and aliquoted to minimize biological variability, each undergoing one freeze–thaw cycle. No additional sample processing occurred between measurements. Therefore, we considered it most appropriate to assess the extent of variation introduced by each acquisition mode without applying intra-mode normalization.

As shown in fig. 2, DIA outperformed the other acquisition modes in the number of detected and MS¹ -annotated features. Its MS² identification yield, however, remained comparable to DDA and AcquireX, all below the typical 10–20 % MS^2^-based identification yield observed for untargeted metabolomics. While DIA theoretically offers broader and unbiased coverage due to its continuous fragmentation of all precursor ions across defined *m/z* windows [29], its performance is closely tied to signal intensity and the ability of the software to deconvolute complex MS² data. In our study, CD3.3 was used for all modes, including DIA. Although CD3.3 enables manual interrogation of features and is well suited for DDA and AcquireX, its limited support for DIA affects the accuracy and depth of MS²-based annotation. Moreover, mzCloud was the only spectral database used for MS² matching, as it is the only one integrated with CD3.3 that supports DIA. While mzCloud is a high-quality, curated library with ∼26,500 compounds [30], it remains not totally comprehensive, and its exclusive use may have limited identification rates across all acquisition modes. Using complementary databases or in-house libraries via mzVault could expand annotation coverage.

While several open-source tools (e.g., MS-DIAL, MZmine) have been developed and benchmarked for untargeted metabolomics workflows—particularly for DIA data—comparing software performance across platforms was beyond the scope of this study and has been done in previous comparative analyses [31], [32], [33]. CD3.3 was selected for data processing as it had already been established in our laboratory workflow prior to introducing DIA and is widely used in Orbitrap-based analyses. It supports reproducible, modular workflows with built-in tools for peak detection, alignment, background subtraction, adduct grouping, and annotation. Compared to some open-source alternatives, CD also offers integrated access to mzCloud, ChemSpider, and KEGG, which allowed us to maintain a consistent database environment across acquisition modes. Another practical advantage was its built-in reporting system, which facilitated communication of instrument methods, processing parameters, and results within our group and with external collaborators. While CD provided sufficient flexibility for DDA and AcquireX analysis, it has limited support for DIA-specific spectral deconvolution, which have contributed to the relatively low number of MS²-based identifications observed in this mode. Moreover, the exclusive use of mzCloud limited annotation coverage. Future studies are certainly benefiting from incorporating alternative software tools and spectral libraries to improve compound identification, particularly for DIA-based workflows in our lab.

Even under highly standardized analytical conditions, we observed considerable variability in metabolite annotation across weeks. For example, DIA showed the highest consistency in identifying the same MS¹ - and MS²-matched features across weeks, yet the overlap of compound names never exceeded 61 % (fig. 3). Several factors unrelated to the acquisition mode likely contributed to this variability. First, small fluctuations in mass accuracy—even within the ± 5 ppm tolerance—can affect database matching, especially when multiple candidate compounds fall within a narrow *m/z* window [26], [34]. Features near the tolerance threshold may be included or excluded depending on the observed mass deviation. Second, even small fluctuations in signal intensity across runs can cause features to intermittently fall above or below peak detection thresholds, resulting in inconsistent feature picking. Third, peak integration and grouping variability—caused by minor retention time shifts or coeluting isomers and/or isobars with *m/z* difference within 5 ppm—can lead to inconsistent feature annotation or grouping across runs. Accurate mass calibration allows narrowing the mass tolerance window down to 1 ppm, substantially reducing variability arising from mass measurement errors. Likewise, rigorous instrument maintenance could further tighten retention time (RT) windows and minimizes variability due to signal fluctuations.

Additionally, we observed cases of incorrect monoisotopic peak or adduct assignment, where features were misidentified due to in-source fragmentation or ion clustering. For example, PGF2α was incorrectly annotated when its molecular ion was attributed to the [M–H₂O–H]⁻ ion of another metabolite (table S14). Similar misassignments have been described by Cooper and Yang (2024) [20], who showed that CD3.3 may interpret [M+H]⁺ ions as neutral losses or misassign adducts, depending on ion detection settings. Such errors can lead to both false positives and negatives, particularly when multiple ions of similar mass are present in complex mixtures. These examples underline the need for caution in interpreting feature-level identifications in untargeted workflows and suggest that database hits—especially MS¹ -based ones—should be treated probabilistically unless supported by orthogonal information. To improve consistency, approaches such as retention time prediction, in silico MS² simulation, in-house library with retention time information for a given *m/z* or orthogonal filtering (e.g., isotope fidelity) could help reduce ambiguity. Moreover, improved handling of adduct and neutral loss relationships in processing software remains a critical area for development.

In addition to acquisition-independent effects, our results underscore key differences in reproducibility and annotation behavior across the three acquisition modes, revealing both strengths and limitations that should be considered when designing untargeted metabolomics studies.

### 4.1 DIA offers high annotation reproducibility, even when incorrect

Among the three modes, DIA showed the highest annotation reproducibility. In several cases, the same compound was consistently annotated with the same name across all three weeks—even though the identification was incorrect. This reflects the deterministic nature of DIA: the same *m/z* isolation windows are scanned in every cycle, generating highly reproducible fragmentation patterns. Although this does not guarantee correct structural assignment, it makes DIA a promising tool when combined with advanced post-processing tools. Our study relied on identity searches within mzCloud, which are currently not compatible with DIA in CD3.3. Therefore, MS² annotation relied on spectral matching limited to direct identity matches. However, if appropriate spectral deconvolution tools (e.g., MS-DIAL or mzmine) were applied, DIA fragmentation could be disentangled based on elution profiles or peak shape correlations, potentially improving the accuracy of annotations and making DIA particularly promising for high-throughput applications. This is particularly important when analyzing different samples. In our case, the same sample was injected on different days leading to the same fragmentation patterns across measurements. However, this might not hold true when analyzing different samples, due to the different elution profiles and spectral deconvolution becomes inevitable.

### 4.2 Stochastic MS² triggering affects reproducibility in DDA and AcquireX

For DDA and AcquireX, reproducibility was hindered by the stochastic nature of MS² acquisition. In several cases, MS² spectra were acquired in one week but not the others, leading to different annotation behaviors. When the MS² scan was available, annotation was based on the fragment spectrum; when absent, the software defaulted to MS¹ -based matching—resulting in inconsistent compound names across weeks (e.g., **Case E** with 5(S),15(S)-DiHETE, table S13). This reflects the well-known limitation of data-dependent methods, where precursor ions must compete for MS² selection and are not always re-fragmented consistently.

### 4.3 Inconsistent annotation due to missing precursor ions

Even when MS² scans were acquired in all weeks, identification could vary due to differences in spectral quality. In several cases (e.g., **Case C** with LTC₄, table S10), DDA spectra did not match their reference in mzCloud despite the presence of a valid reference spectrum. Visual inspection revealed that the precursor ion was missing from the MS² scan, likely due to off-apex fragmentation, low precursor intensity, wrong MS^1^- MS^2^ grouping, or complete dissociation under the collision energies used (HCD 30–50). Since we relied on identity searches—requiring the presence of precursor ions—such spectra were not matched to any spectrum. This conservative approach reduced overannotation but also reduced identification rates. Similarity search, which uses cosine similarity to detect related compounds, is available in CD for DDA and AcquireX but not for DIA, and was not used here to maintain comparability. The reliance on identity search, while stringent, reduces the potential MS²-based identifications for DDA and AcquireX and explains the low MS^2^-based identification rate observed for all acquisition modes.

### 4.4 Fragmentation pattern variability for the same compound

We also observed cases in which the same eicosanoid standard yielded different fragmentation patterns across weeks using DDA. For example, in **Case D**, 6-keto-PGF1α and 8-iso-15-keto-PGE2 (figures S11, S12) showed distinct spectral profiles over time, leading to different annotations or lack of matching in some weeks. These differences may stem from variation in MS^2^-sampling along the elution profile, coelution with other metabolic features, or instrument fluctuations. However, they also raise concerns about the stability of MS¹ –MS² pairing in DDA (also valid for point 4.3 above). While DDA is generally assumed to preserve the link between precursor and product ions, our observations suggest that this assumption does not always hold. Misassignment of precursor ions during processing—either due to incorrect peak picking or overlapping features—may lead to fragment spectra being attributed to the wrong parent ion. A similar concern was raised by Cooper & Yang (2024), who observed that in AcquireX datasets, MS^2^ spectra representing two different isomers can get grouped with a single parent, resulting in false identifications [20]. This suggests that the presumed superiority of DDA in maintaining precursor–fragment linkage requires re-evaluation, especially when using semi-automated or automated annotation pipelines.

The persistent misannotation of spiked eicosanoid standards lacking reference spectra in mzCloud, as well as the observed low-confidence or inconsistent MS² matches across acquisition modes (**cases A and B**), highlights a broader structural limitation: the lack of comprehensive, standardized, and high-quality MSⁿ spectral libraries. This issue extends beyond our study design and reflects a critical bottleneck in untargeted metabolomics. Without sufficient spectral coverage for known biologically relevant compounds, particularly for structurally similar lipids and isomers, even high-quality MS² data cannot yield confident identifications. Standardizing MS² acquisition parameters and expanding curated, spectral databases—especially for negative-ion mode and soft fragmentation conditions—are essential for improving annotation reliability across studies.

These findings highlight the importance of acquisition-aware data processing strategies. For DIA, the observed consistency in fragmentation patterns suggests strong potential for reproducible annotation—particularly if future studies apply spectral deconvolution tools that can better assign fragment ions based on peak shape or chromatographic behavior. While such tools (e.g., MS-DIAL and mzmine) exist, their integration into routine workflows, especially in regulated or platform-specific environments, is still limited. For DDA and AcquireX, addressing the variability in MS² acquisition is more challenging. One possibility is to optimize acquisition parameters—such as reducing cycle times or adjusting dynamic exclusion settings—to increase MS² coverage and improve consistency. However, this often comes at the cost of reduced scan quality or duty cycle limitations, so any changes must be carefully evaluated depending on study goals. Incorporating similarity searches alongside identity matching may help improve annotation rates, but also introduces the risk of overannotation and requires careful validation. While we did not use similarity scoring in this study to maintain stringency and comparability, it remains a potentially useful complementary approach, particularly for exploratory analyses. The use of matrix-matched QC samples spiked with known standards could also help monitor consistency of MS² acquisition across sequences. However, this is only effective if the standards are well-behaved and span the chemical diversity of interest—something that remains a limitation for many lipid or polar metabolite classes. Manual review of fragment spectra remains the most reliable way to confirm uncertain annotations, especially for compounds of biological interest, but is not scalable for large datasets.

While our use of CD3.3 may limit the generalizability of some findings to other platforms, it reflects the reality for many users operating within Orbitrap-based workflows. One of the many advantages offered by CD3.3 is its ability to expose multiple layers of data—including raw spectra, precursor-product pairing, and match scores—which allows for detailed interrogation of identification outcomes. Therefore, we strongly encourage users of CD3.3 to engage with this functionality and not rely solely on top-ranked hits or match scores. A high match score does not guarantee a correct annotation, particularly when underlying issues like adduct misassignment, missing precursor ions, or inconsistent fragmentation are present. Instead, annotations should be evaluated across multiple replicates to assess their robustness. Moreover, since the matching logic for DDA (and AcquireX) is broadly similar across software platforms, we believe that many of the annotation inconsistencies observed here may also occur using other tools—making these findings relevant beyond CD3.3 alone.

Another challenge observed in our dataset was the misannotation of coeluting isomers, particularly PGD2 and PGF3α. In our chromatographic setup, these two compounds consistently coeluted, producing a single chromatographic peak producing mixed MS^2^ spectra (table S14). When analyzed in untargeted mode, the peak was annotated as PGF3α across all acquisition modes (fig. 4, green circle). The preferential annotation of PGF3α might stem from its higher ionization efficiency and more dominant fragmentation signature, resulting in a stronger match score with the PGF3α MS² reference spectrum. Because these isomers have identical precursor *m/z* values and highly similar fragmentation patterns, the lack of chromatographic separation and reliance on spectral similarity leads to systematic misannotation. This is a well-documented limitation in untargeted metabolomics, particularly for structural isomers and lipid mediators. When chromatographic resolution is insufficient, even high-resolution MS² data cannot reliably distinguish such compounds. As discussed by Schrimpe-Rutledge et al., potential solutions include the use of orthogonal separation strategies, such as two-dimensional chromatography or ion mobility spectrometry (IMS), which offer orthogonal separation based on polarity or molecular shape, respectively [35]. However, these approaches increase analytical complexity and are not always accessible. Alternatively, using known standards to validate retention time and fragmentation behavior in targeted follow-up experiments can help resolve ambiguous features—although this shifts interpretation toward a semi-targeted workflow. In our case, targeted separation strategies would have been necessary to confirm the presence of PGD₂ alongside PGF3α. This example illustrates how isomer coelution can mislead annotation in untargeted workflows and underscores the value of combining orthogonal evidence—chromatographic, spectral, and quantitative—to support reliable compound identification.

The third aim of our study was to compare DIA, DDA, and AcquireX in terms of their detection power for low-abundance metabolites, using a dilution series of 14 eicosanoid standards spiked into TLE samples. Under comparable MS conditions, all acquisition modes successfully identified the spiked eicosanoids as upregulated at the highest concentration (10 ng/mL). At a 10-fold lower concentration (1 ng/mL), DIA demonstrated slightly better detection performance, capturing 12 standards, compared to 11 detected by DDA and AcquireX, respectively. However, at lower concentrations (0.1 ng/mL and 0.01 ng/mL), DIA’s performance declined. This was likely due to its longer cycle time (∼3 s for 33 windows at 12 Hz), which results in fewer full scans across chromatographic peaks and consequently lower signal-to-noise ratios and/or badly shaped peaks that get sorted out during processing. In our setup, this cycle time yielded approximately 7–10 data points per peak—sufficient to ensure quantification and reproducibility under the chromatographic conditions used, as recommended in standard guidelines [36]. The high number of DIA windows was selected to increase precursor coverage and support improved MS² fragmentation quality. In contrast, DDA and AcquireX operated at shorter cycle times (∼0.6 s) and benefited from lower intensity thresholds (10^3^), which favored the triggering of MS² scans for low-abundance compounds. This likely contributed to their relatively better performance at the lowest concentrations, although with high variability in detection. Additionally, the performance of each acquisition mode can be further optimized toward specific goals—whether maximizing sensitivity, MS² coverage, or even reproducibility. These findings reinforce a broader principle in untargeted metabolomics: the absence of detection should not be equated with the absence of a metabolite. Detection sensitivity is highly dependent on acquisition parameters, compound-specific properties, and post-processing strategies. A fair comparison across acquisition strategies should therefore consider the intended analytical objective and the specific trade-offs inherent to each method.

While these results provide insight into the trade-offs between MS^2^ coverage and sensitivity, it is important to note that our comparison is limited by the small number of analytes and their shared chemical class. The inclusion of only 14 eicosanoid standards does not allow broad generalization to other metabolite classes with differing physicochemical properties. While other approaches, like matrix dilution series, are widely used to assess sensitivity across diverse compounds, our approach was deliberately chosen to mimic realistic biomarker discovery conditions, where complex biological matrices are typically not diluted. By spiking a well-characterized and structurally diverse set of eicosanoids into a constant matrix background, we aimed to evaluate the relative performance of each acquisition method in detecting and identifying low-abundance, biologically relevant features under comparable and controlled conditions. Although this targeted selection allowed us to rigorously assess detection sensitivity and MS^2^-based identification accuracy, we recognize that expanding the analysis to include a broader metabolite panel and matrix dilution series would provide complementary insights.

## 5 Conclusion

This study presents a systematic, side-by-side comparison of three commonly used acquisition strategies—DIA, DDA, and AcquireX—for untargeted LC-HRAM-MS/MS-based metabolomics, evaluated across multiple dimensions including reproducibility, identification accuracy, and detection sensitivity at low concentrations. By applying standardized MS conditions and data processing workflow in Compound Discoverer, we were able to isolate acquisition-specific sources of variability and assess their impact under realistic, multi-day analytical conditions.

Daily system suitability tests (SSTs) proved essential for assessing instrument readiness, helping us monitor critical parameters such as retention time, mass accuracy, and peak area reproducibility. These checks were instrumental in ensuring that untargeted measurements were performed only when the system met predefined performance criteria. However, our findings also reveal that SSTs alone are not sufficient to capture subtler, sample-specific sources of variability—such as signal drift, ion suppression, or changes in fragmentation behavior over time. For this reason, matrix-matched or pooled QC samples remain a necessary complement to SSTs. Regular injection of such QCs across sequences enables the monitoring and correction of intra- and inter-batch variability, supports post-acquisition normalization, and facilitates quality control of feature detection and annotation consistency.

Our findings demonstrate that DIA offers the highest reproducibility in both feature counts and structural annotation, although it remains constrained by limited MS² identification support in conventional software pipelines. In contrast, DDA and AcquireX showed greater variability in both MS² acquisition and spectral annotation, stemming from their stochastic fragmentation behavior and limited sampling of low-abundance compounds. We also observed acquisition mode-independent sources of variability—including mass accuracy fluctuations, adduct misassignments, and coelution of isomeric compounds—which further challenge annotation stability, even under standardized conditions. Looking at the sensitivity assessment, a general cut-off was observed for the three acquisition modes. None of this assessed acquisition modes was able to detect and/or identify eicosanoids at the physiological level, explaining why they are usually overlooked during untargeted analyses. Detection of specific classes of metabolites, especially low abundance ones, may require further optimization in analytical conditions or enrichment during pre-analytical steps.

It is equally important to recognize that method performance cannot be fairly compared without considering the optimization priorities of each workflow. In our case, acquisition parameters were tuned to maximize MS² coverage rather than sensitivity, particularly for DIA, which helps explain its limited performance at the lowest concentration levels. While the use of 14 eicosanoid standards restricts broad generalization, the study provides important insight into the inherent trade-offs between MS^2^ coverage, sensitivity, and reproducibility. Crucially, these findings reinforce a broader principle in untargeted metabolomics: a non-detected metabolite cannot be equated with its absence, and high-confidence identification should be supported by multiple orthogonal criteria rather than a single match score.

Looking forward, the continued development of more comprehensive and chemically diverse spectral libraries, improved handling of MS² quality in data processing, and integrated QC-driven normalization workflows will be critical for improving the robustness and transparency of untargeted metabolomics pipelines. In the meantime, users are encouraged to remain cautious in interpreting automated annotations—particularly for isomers or low-abundance features—and to critically evaluate the assumptions embedded in both acquisition strategies and software tools.

## CRediT authorship contribution statement

**Hanane El Boudlali:** Writing – review & editing, Writing – original draft, Visualization, Validation, Methodology, Investigation, Formal analysis, Data curation, Conceptualization. **Uta Ceglarek:** Writing – review & editing, Supervision, Resources, Funding acquisition, Conceptualization. **Laura Lehmicke:** Writing – review & editing, Visualization, Validation, Investigation, Formal analysis.

## Associated content

The supporting information is available for review and publication.

## Funding

This research was funded by a grant from the Deutsche Forschungsgemeinschaft (10.13039/100004807DFG, German Research Foundation)—Project number 209933838—Collaborative Research Center SFB1052 “Obesity Mechanisms”, to U.C. (SFB-1052/A9).

## Declaration of Competing Interest

The authors declare that they have no known competing financial interests or personal relationships that could have appeared to influence the work reported in this paper.

## References

[1] Winson Oliver S.G., Kell M.K., Baganz D.B. (1998). F. Systematic functional analysis of the yeast genome. Trends Biotechnol.

[2] Fiehn O., Kopka J., Dörmann P., Altmann T., Trethewey R.N., Willmitzer L. (2000). Metabolite profiling for plant functional genomics. Nat Biotechnol.

[3] Ambati C.S.R., Yuan F., Abu-Elheiga L.A., Zhang Y., Shetty V. (2017). Identification and quantitation of malonic acid biomarkers of in-born error metabolism by targeted metabolomics. J Am Soc Mass Spectrom.

[4] Klein M.S., Shearer J. (2016). Metabolomics and type 2 diabetes: translating basic research into clinical application. J Diabetes Res.

[5] Wang X., Chen S., Jia W. (2016). Metabolomics in cancer biomarker research. Curr Pharm Rep.

[6] Zhang A., Sun H., Yan G., Wang P., Wang X. (2016). Mass spectrometry-based metabolomics: applications to biomarker and metabolic pathway research. Biomed Chromatogr.

[7] Ren S., Shao Y., Zhao X., Hong C.S., Wang F., Lu X. (2016). Integration of metabolomics and transcriptomics reveals major metabolic pathways and potential biomarker involved in prostate cancer. Mol Cell Proteom.

[8] Würtz P., Cook S., Wang Q., Tiainen M., Tynkkynen T., Kangas A.J. (2016). Metabolic profiling of alcohol consumption in 9778 young adults. Int J Epidemiol.

[9] Kortz L., Dorow J., Becker S., Thiery J., Ceglarek U. (2013). Fast liquid chromatography-quadrupole linear ion trap-mass spectrometry analysis of polyunsaturated fatty acids and eicosanoids in human plasma. J Chromatogr B Anal Technol Biomed Life Sci.

[10] Ortmayr K., Causon T.J., Hann S., Koellensperger G. (2016). Increasing selectivity and coverage in LC-MS based metabolome analysis. TrAC Trends Anal Chem.

[11] Fekete S., Veuthey J.-L., Guillarme D. (2015). Comparison of the most recent chromatographic approaches applied for fast and high resolution separations: theory and practice. J Chromatogr A.

[12] Wolfender J.-L., Nuzillard J.-M., van der Hooft J.J.J., Renault J.-H., Bertrand S. (2019). Accelerating metabolite identification in natural product research: toward an ideal combination of liquid chromatography-high-resolution tandem mass spectrometry and NMR profiling, in silico databases, and chemometrics. Anal Chem.

[13] Böcker S., Letzel M.C., Lipták Z., Pervukhin A. (2009). SIRIUS: decomposing isotope patterns for metabolite identification. Bioinformatics.

[14] Broeckling C.D., Hoyes E., Richardson K., Brown J.M., Prenni J.E. (2018). Comprehensive tandem-mass-spectrometry coverage of complex samples enabled by data-set-dependent acquisition. Anal Chem.

[15] Reisdorph N.A., Walmsley S., Reisdorph R. (2019). A perspective and framework for developing sample type specific databases for LC/MS-based clinical metabolomics. Metabolites.

[16] Sumner L.W., Amberg A., Barrett D., Beale M.H., Beger R., Daykin C.A. (2007). Proposed minimum reporting standards for chemical analysis Chemical Analysis Working Group (CAWG) Metabolomics Standards Initiative (MSI). Metabolomics.

[17] Benton H.P., Ivanisevic J., Mahieu N.G., Kurczy M.E., Johnson C.H., Franco L. (2015). Autonomous metabolomics for rapid metabolite identification in global profiling. Anal Chem.

[18] Perez de Souza L., Alseekh S., Scossa F., Fernie A.R. (2021). Ultra-high-performance liquid chromatography high-resolution mass spectrometry variants for metabolomics research. Nat Methods.

[19] Guo J., Huan T. (2020). Comparison of full-scan, data-dependent, and data-independent acquisition modes in liquid chromatography-mass spectrometry based untargeted metabolomics. Anal Chem.

[20] Cooper B., Yang R. (2024). An assessment of AcquireX and compound discoverer software 3.3 for non-targeted metabolomics. Sci Rep.

[21] Wang D., DuBois R.N. (2007). Measurement of eicosanoids in cancer tissues. Methods Enzym.

[22] Kortz L., Dorow J., Ceglarek U. (2014). Liquid chromatography-tandem mass spectrometry for the analysis of eicosanoids and related lipids in human biological matrices: a review. J Chromatogr B Anal Technol Biomed Life Sci.

[23] Sheppe A.E.F., Edelmann M.J. (2021). Roles of eicosanoids in regulating inflammation and neutrophil migration as an innate host response to bacterial infections. Infect Immun.

[24] Quehenberger O., Armando A.M., Brown A.H., Milne S.B., Myers D.S., Merrill A.H. (2010). Lipidomics reveals a remarkable diversity of lipids in human plasma. J Lipid Res.

[25] Gomolka B., Siegert E., Blossey K., Schunck W.-H., Rothe M., Weylandt K.H. (2011). Analysis of omega-3 and omega-6 fatty acid-derived lipid metabolite formation in human and mouse blood samples. Prostaglandins Other Lipid Mediat.

[26] Broadhurst D., Goodacre R., Reinke S.N., Kuligowski J., Wilson I.D., Lewis M.R. (2018). Guidelines and considerations for the use of system suitability and quality control samples in mass spectrometry assays applied in untargeted clinical metabolomic studies. Metabolomics.

[27] Dunn W.B., Broadhurst D., Begley P., Zelena E., Francis-McIntyre S., Anderson N. (2011). Procedures for large-scale metabolic profiling of serum and plasma using gas chromatography and liquid chromatography coupled to mass spectrometry. Nat Protoc.

[28] Yue Y., Bao X., Jiang J., Li J. (2022). Evaluation and correction of injection order effects in LC-MS/MS based targeted metabolomics. J Chromatogr B Anal Technol Biomed Life Sci.

[29] Wolfender J.-L., Marti G., Thomas A., Bertrand S. (2015). Current approaches and challenges for the metabolite profiling of complex natural extracts. J Chromatogr A.

[30] re3data.org. mzCloud: re3data.org - Registry of Research Data Repositories; 2017. 10.1371/journal.pone.0078080 PMC3817176 24223762

[31] Wartmann Y., Boxler M.I., Kraemer T., Steuer A.E. (2024). Impact of three different peak picking software tools on the quality of untargeted metabolomics data. J Pharm Biomed Anal.

[32] Aigensberger M., Bueschl C., Castillo-Lopez E., Ricci S., Rivera-Chacon R., Zebeli Q. (2025). Modular comparison of untargeted metabolomics processing steps. Anal Chim Acta.

[33] Wang X.-C., Ma X.-L., Liu J.-N., Zhang Y., Zhang J.-N., Ma M.-H. (2023). A comparison of feature extraction capabilities of advanced UHPLC-HRMS data analysis tools in plant metabolomics. Anal Chim Acta.

[34] Kind T., Fiehn O. (2007). Seven golden rules for heuristic filtering of molecular formulas obtained by accurate mass spectrometry. BMC Bioinforma.

[35] Schrimpe-Rutledge A.C., Codreanu S.G., Sherrod S.D., McLean J.A. (2016). Untargeted metabolomics strategies-challenges and emerging directions. J Am Soc Mass Spectrom.

[36] Zeng W., Bateman K.P. (2023). Quantitative LC-MS/MS. 1. Impact of points across a peak on the accuracy and precision of peak area measurements. J Am Soc Mass Spectrom.

