# Supplementary material for: High-resolution accurate mass- mass spectrometry based- untargeted metabolomics: Reproducibility and detection power across data-dependent acquisition, data-independent acquisition, and AcquireX
Source: Comput Struct Biotechnol J. 2025 May 30;27:2412–23. doi: 10.1016/j.csbj.2025.05.046 (PMC12173630; doi:10.1016/j.csbj.2025.05.046)
Supplement: Supplementary file 1 — Supplementary material [file mmc1.docx]

Supporting information

For *Computational and Structural Biotechnology Journal*

Authors

Hanane El Boudlali, Laura Lehmicke, Uta Ceglarek

**High-Resolution Accurate Mass- Mass Spectrometry based- Un-targeted metabolomics: reproducibility and detection power across Data-Dependent Acquisition, Data-Independent Acquisition, and AcquireX**

# Detailed methods

## Supplemental table S1. Chemical structures and physical properties of the selected eicosanoid representatives.

| Tetranor-Prostaglandin D Metabolite (PGDM)   \| *Structure* \| 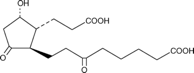 \| \| --- \| --- \| \| *CAS number* \| 70803-91-7 \| \| *Molecular formula* \| C16H24O7 \| \| *Molecular weight (g/mol)* \| 328.4 \| \|  \|  \| | \| *Grade* \| Analytical standard, Cayman Chemical \| \| --- \| --- \| |
| --- | --- | --- | --- | --- | --- | --- | --- | --- | --- | --- | --- | --- | --- |
|  | \| *Purity* \| ≥90% \| \| --- \| --- \| \| *pKa^a^* \| 3.97 \| \| *log P^b^* \| 1.70 \| |
| 20-hydroxy Prostaglandin E_2_ (PGE2)   \| *Structure* \| 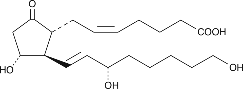 \| \| --- \| --- \| \| *CAS number* \| 57930-95-7 \| \| *Molecular formula* \| C20H32O6 \| \| *Molecular weight (g/mol)* \| 368.5 \| \|  \|  \| | \|  \|  \| \| --- \| --- \| \|  \|  \| \| *Grade* \| Analytical standard, Cayman Chemical \| \| *Purity* \| ≥95% \| \| *pKa^a^* \| 4.3, -1.47 \| \| *log P^b^* \| 3.08 \| |
| *6-keto Prostaglandin F1α*   \| *Structure* \| 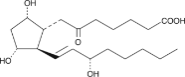 \| \| --- \| --- \| \| *CAS number* \| 58962-34-8 \| \| *Molecular formula* \| C20H34O6 \| \| *Molecular weight (g/mol)* \| 370.5 \| \|  \|  \| | \| *Grade* \| Analytical standard, Cayman Chemical \| \| --- \| --- \|  \| *Purity* \| ≥98% \| \| --- \| --- \| \| *pKa^a^* \| 4.14; -1.63 \| \| *log P^b^* \| 3.31 \| |
| *Prostaglandin F_3α_ (PF3α)*   \| *Structure* \| 1,2  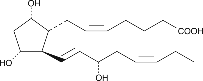 \| \| --- \| --- \| \| *CAS number* \| 745-64-2 \| \| *Molecular formula* \| C20H32O5 \| \| *Molecular weight (g/mol)* \| 352.5 \| \|  \|  \| | \| *Grade* \| Analytical standard, Cayman Chemical \| \| --- \| --- \|  \| *Purity* \| ≥98% \| \| --- \| --- \| \| *pKa^a^* \| 4.36; -1.67 \| \| *log P^b^* \| 3.68 \| |
| Prostaglandin F_2α_ (PGF2α)   \| *Structure* \| 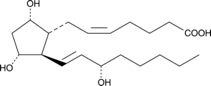 \| \| --- \| --- \| \| *CAS number* \| 551-11-1 \| \| *Molecular formula* \| C20H34O5 \| \| *Molecular weight (g/mol)* \| 354.5 \| \|  \|  \| | \|  \|  \| \| --- \| --- \| \| *Grade* \| Analytical standard, Cayman Chemical \|  \| *Purity* \| ≥98% \| \| --- \| --- \| \| *pKa^a^* \| 4.36; -1.63 \| \| *log P^b^* \| 3.90 \| |
| Prostaglandin D_2_ (PGD2)   \| *Structure* \| 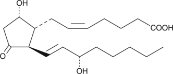 \| \| --- \| --- \| \| *CAS number* \| C20H32O5 \| \| *Molecular formula* \| C_13_H_16_O_4_ \| \| *Molecular weight (g/mol)* \| 352.5 \| \|  \|  \| | \| *Grade* \| Analytical standard, Cayman Chemical \| \| --- \| --- \|  \| *Purity* \| ≥98% \| \| --- \| --- \| \| *pKa^a^* \| 4.4; -1.6 \| \| *log P^b^* \| 3.82 \| |
| 8-iso-15-keto Prostaglandin E_2_ (PGE2)   \| *Structure* \| 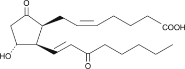 \| \| --- \| --- \| \| *CAS number* \| 914804-63-0 \| \| *Molecular formula* \| C20H30O5 \| \| *Molecular weight (g/mol)* \| 350.5 \| \|  \|  \| | \| *Grade* \| Analytical standard, Cayman Chemical \| \| --- \| --- \|  \| *Purity* \| ≥98% \| \| --- \| --- \| \| *pKa^a^* \| 4.3 \| \| *log P^b^* \| 3.75 \| |
| Leukotrine C_4_  (LTC4)   \| *Structure* \| 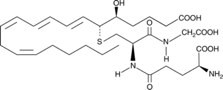 \| \| --- \| --- \| \| *CAS number* \| 72025-60-6 \| \| *Molecular formula* \| C30H47N3O9S \| \| *Molecular weight (g/mol)* \| 625.8 \| \|  \|  \| | \| *Grade* \| Analytical standard, Cayman Chemical \| \| --- \| --- \|  \| *Purity* \| ≥95 \| \| --- \| --- \| \| *pKa^a^* \| 1.71; 9.31 \| \| *log P^b^* \| 4.49 \| |
| 5(S),15(S)-DiHETE   \| *Structure* \| 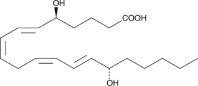 \| \| --- \| --- \| \| *CAS number* \| 82200-87-1 \| \| *Molecular formula* \| C20H32O4 \| | \| *Molecular weight (g/mol)* \| 336.5 \| \| --- \| --- \|  \| *Grade* \| Analytical standard, Cayman Chemical \| \| --- \| --- \|  \| *Purity* \| ≥95% \| \| --- \| --- \| \| *pKa^a^* \| 4.58; -1.26 \| \| *log P^b^* \| 4.73 \| |
| 12(S)-HHT   \| *Structure* \| 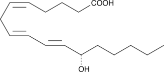 \| \| --- \| --- \| \| *CAS number* \| 54397-84-1 \| \| *Molecular formula* \| C17H28O3 \| \| *Molecular weight (g/mol)* \| 280.4 \| \|  \|  \| | \| *Grade* \| Analytical standard, Cayman Chemical \| \| --- \| --- \|  \| *Purity* \| ≥95% \| \| --- \| --- \| \| *pKa^a^* \| 4.89; -1.58 \| \| *log P^b^* \| 4.53 \| |
| 12(S)-HEPE   \| *Structure* \| 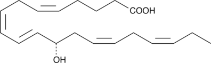 \| \| --- \| --- \| \| *CAS number* \| 116180-17-7 \| \| *Molecular formula* \| C20H30O3 \| \| *Molecular weight (g/mol)* \| 318.5 \| \|  \|  \| | \| *Grade* \| Analytical standard, Cayman Chemical \| \| --- \| --- \|  \| *Purity* \| ≥98% \| \| --- \| --- \| \| *pKa^a^* \| 4.89; -1.61 \| \| *log P^b^* \| 5.25 \| |
| 15(S)-HETE   \| *Structure* \| 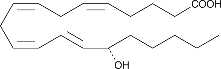 \| \| --- \| --- \| \| *CAS number* \| 54845-95-3 \| \| *Molecular formula* \| C20H32O3 \| \| *Molecular weight (g/mol)* \| 320.5 \| \|  \|  \| | \| *Grade* \| Analytical standard, Cayman Chemical \| \| --- \| --- \| \| *pKa^a^* \| 4.82; -1.58 \| \| *log P^b^* \| 5.47 \| |
| 12-oxo-ETE   \| *Structure* \| 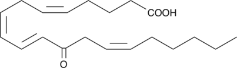 \| \| --- \| --- \| \| *CAS number* \| 108437-64-5 \| \| *Molecular formula* \| C20H30O3 \| \| *Molecular weight (g/mol)* \| 318.5 \| \|  \|  \| | \| *Grade* \| Analytical standard, Cayman Chemical^c^ \| \| --- \| --- \|  \| *Purity* \| ≥90% \| \| --- \| --- \| \| *pKa^a^* \| 4.89 \| \| *log P^b^* \| 5.40 \| |
| (+/-)8,9-EET   \| *Structure* \| 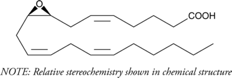 \| \| --- \| --- \| \| *CAS number* \|  \| \| *Molecular formula* \| C20H32O3 \| \| *Molecular weight (g/mol)* \| 320.5 \| \|  \|  \| | \| *Grade* \| Analytical standard, Cayman Chemical \| \| --- \| --- \|  \| *Purity* \| ≥98% \| \| --- \| --- \| \| *pKa^a^* \| 4.46 \| \| *log P^b^* \| 6.00 \| |

^a^ Adapted from reference (www.chemicalize.org (accessed 24.06.2024)). Only strongest acidic or Only strongest acidic or basic pKa values are shown.

^b^ Adapted from LIPID MAPS (<https://www.lipidmaps.org/> (accessed 08.05.2024))

^c^ Cayman Chemical (Michigan, USA)

DiHETE, dihydroxyeicosatrienoic acid; HHT, hydroxyheptadecatrienoic; HETE, hydroxy-eicosatetraenoic acid; HEPE,

hydroxyeicosapentaenoic acid; ETE, eicosatetraenoic acid; EET, epoxyeicosatrienoic acid

## Supplemental table S2: m/z Windows used during vDIA acquisition.

| **m/z range** | **m/z range** |
| --- | --- |
| 180-240 | 540-560 |
| 240-260 | 560-580 |
| 260-280 | 580-600 |
| 280-300 | 600-620 |
| 300-320 | 620-640 |
| 320-340 | 640-660 |
| 340-360 | 660-680 |
| 360-380 | 680-700 |
| 380-400 | 700-750 |
| 400-420 | 750-800 |
| 420-440 | 800-850 |
| 440-460 | 850-900 |
| 460-480 | 950-1000 |
| 480-500 | 1000-1050 |
| 500-520 | 1050-1100 |
| 520-540 | 1100-1150 |
| 1150-1200 | |

## Supplemental **table S3:** Chromatographic range parameters used in Freestyle^TM^ for evaluation of the system suitability test.

| **Standard** | **Filter** | **Chemical Formula** | **Ranges** | **Trace Type** | **Smoothing** | **Detector Type** | **Mass Tolerance** |
| --- | --- | --- | --- | --- | --- | --- | --- |
| Tetranor-PGDM | FTMS - p ESI Full ms [180.0000-1200.0000] | C16H24O7 | 327.1449 | Mass Range | None | MS | ± 5 ppm |
| 20-hydroxy-PGE2 | FTMS - p ESI Full ms [180.0000-1200.0000] | C20H32O6 | 367.2126 | Mass Range | None | MS | ± 5 ppm |
| 6-keto-PGF1a | FTMS - p ESI Full ms [180.0000-1200.0000] | C20H34O6 | 369.2283 | Mass Range | None | MS | ± 5 ppm |
| PGF3a | FTMS - p ESI Full ms [180.0000-1200.0000] | C20H32O5 | 351.2177 | Mass Range | None | MS | ± 5 ppm |
| PGD2 | FTMS - p ESI Full ms [180.0000-1200.0000] | C20H32O5 | 351.2177 | Mass Range | None | MS | ± 5 ppm |
| TxB1 | FTMS - p ESI Full ms [180.0000-1200.0000] | C20H36O6 | 371.2439 | Mass Range | None | MS | ± 5 ppm |
| 8-iso-15-keto-PGE2 | FTMS - p ESI Full ms [180.0000-1200.0000] | C20H30O5 | 349.202 | Mass Range | None | MS | ± 5 ppm |
| PGF2a | FTMS - p ESI Full ms [180.0000-1200.0000] | C20H34O5 | 353.2333 | Mass Range | None | MS | ± 5 ppm |
| LTC4 | FTMS - p ESI Full ms [180.0000-1200.0000] | C30H47N3O9S | 624.296 | Mass Range | None | MS | ± 5 ppm |
| 5(S),15(S)-DiHETE | FTMS - p ESI Full ms [180.0000-1200.0000] | C20H32O4 | 335.2228 | Mass Range | None | MS | ± 5 ppm |
| 12(S)-HHT | FTMS - p ESI Full ms [180.0000-1200.0000] | C17H28O3 | 279.1966 | Mass Range | None | MS | ± 5 ppm |
| 12(S)-HEPE | FTMS - p ESI Full ms [180.0000-1200.0000] | C20H30O3 | 317.2122 | Mass Range | None | MS | ± 5 ppm |
| 15(S)-HETE | FTMS - p ESI Full ms [180.0000-1200.0000] | C20H32O3 | 319.2279 | Mass Range | None | MS | ± 5 ppm |
| 12-oxo-ETE | FTMS - p ESI Full ms [180.0000-1200.0000] | C20H30O3 | 317.2122 | Mass Range | None | MS | ± 5 ppm |
| (+/-)8,9-EpETrE | FTMS - p ESI Full ms [180.0000-1200.0000] | C20H32O3 | 319.2279 | Mass Range | None | MS | ± 5 ppm |

##
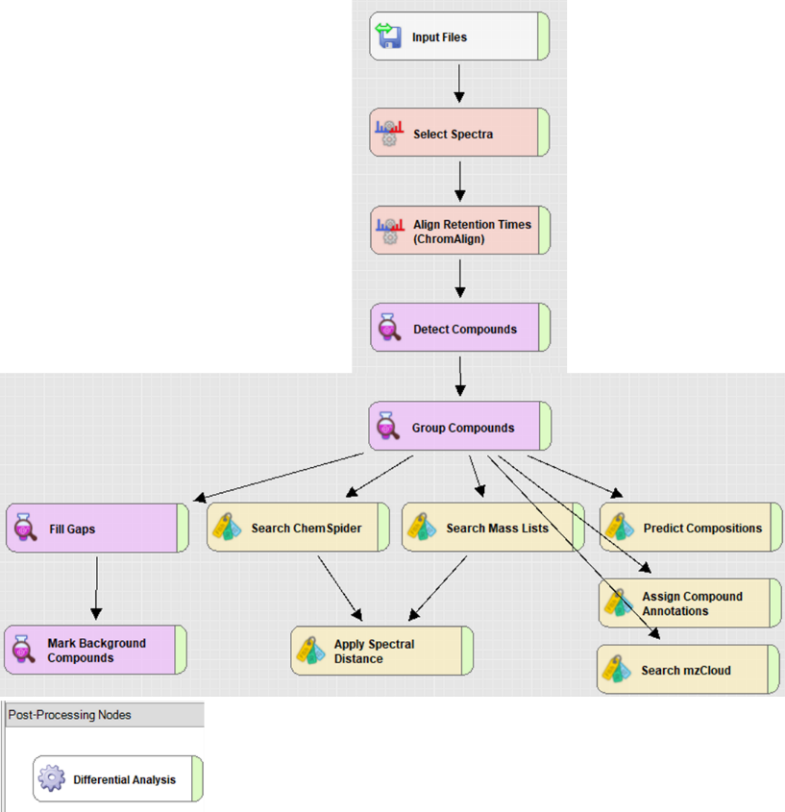
**Supplemental figure S1:** Compound Discoverer workflow – used nodes and connection overview.


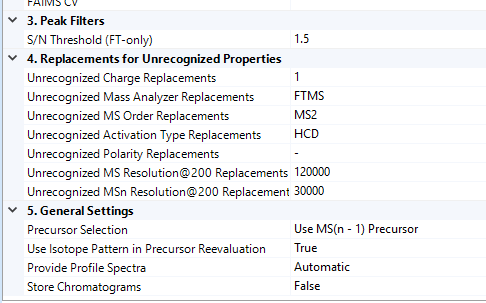

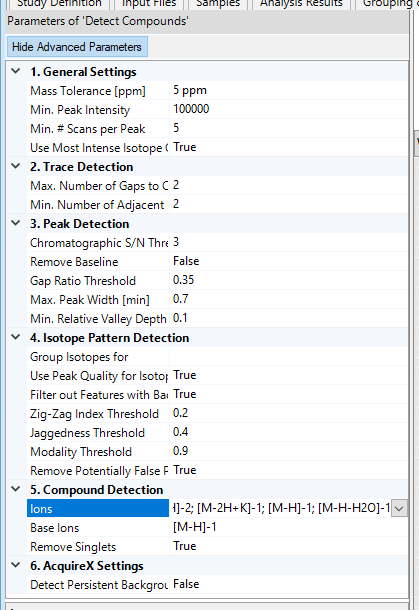

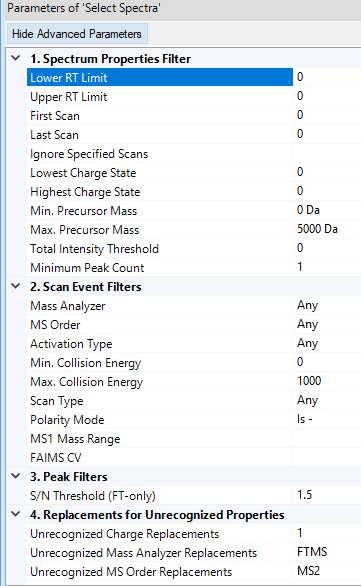

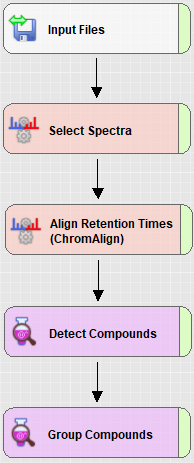

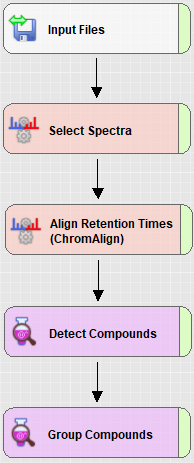

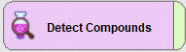

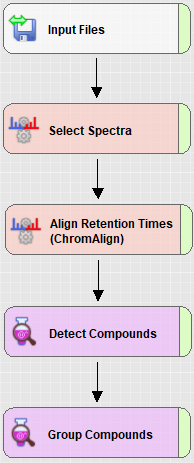

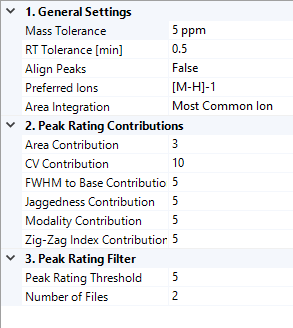


All the files are aligned to reference file specified here. If not, the first QC or first sample file is taken as reference.


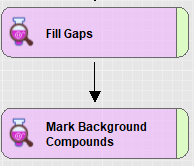

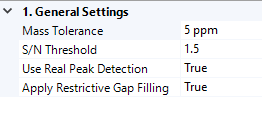

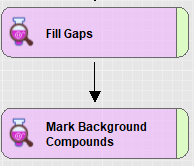

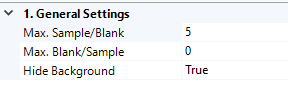


b

a

## **Supplemental figure S2:** Compound Discoverer workflow parameters (part 1) for untargeted compound identification.

^a^ Ions selected for detection: [2M+FA-H]^-1^, [2M-H]^-1^, [M+FA-H]^-1^, [M-2H]^-2^, [M-2H+K]^-1^, [M-H-H_2_O]^-1^, [M-H]^-1^.

^b^ Set as true when analyzing AcquireX data, otherwise false.


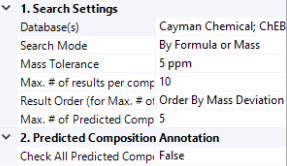

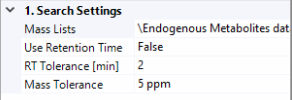

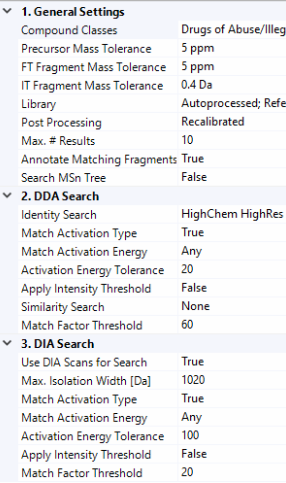

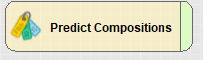

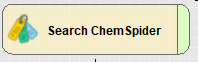

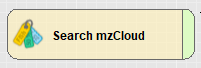

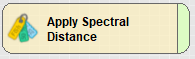

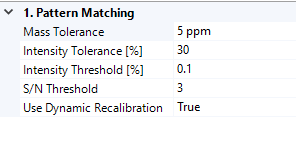

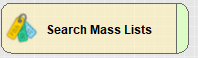

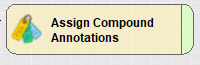

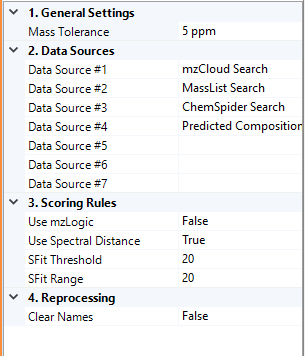


b

c

a

d


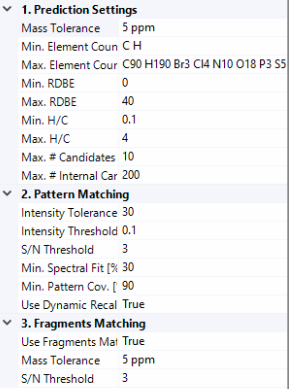


e

## **Supplemental figure S3:** Compound Discoverer workflow parameters (part 2, continued) for untargeted compound identification.

a Compound classes: Drugs of Abuse/Illegal Drugs; Endogenous Metabolites; Natural Products/Medicines; Natural Toxins; Perfluorinated Hydrocarbons; Steroids/Vitamins/Hormones; Therapeutics/Prescription Drugs.

^b^ Libraries: Autoprocessed and Reference.

^c^ Set as true when analyzing data acquired with DIA, false otherwise.

^d^ Local mass lists: Endogenous Metabolites database 4400 compounds , Natural Products Atlas (last update 08.2021), LMSD structure data file (last update; 26.03.2024).

^e^ Databases: Cayman Chemical; ChEBI; Human Metabolome Database; KEGG; LIPID MAPS; Serum Metabolome Database; SMPDB Small Molecule Pathway Database.

## **Supplemental table S4:** Statistical methods applied, including descriptions and purposes.

| Node | Processing step | Method description |
| --- | --- | --- |
| Align retention time (ChromAlign) | Retention Time Alignement | Retention time alignement performed by the ChromAlign algorithm: Sadygov et al. (https://doi.org/10.1021/ac060923y) |
| Group Compounds | [Compounds] Apply Peak Rating Filter | Only compounds with an Original Peak Rating greater or equal to 5 in at least 2 samples are kept for further processing |
| Fill Gaps | Similar Features Search  Centroids Filtering  Detection | Features search within a tolerance of 5 ppm  Filtered centroids with S/N threshold = 1.5  Real detection (for more accurate areas) was performed |
| ^a^Differential Analysis (Post-processing node) | Peak Rating Contribution  [Compounds] Input Data  [Compounds] Data Transformation  [Compounds] Statistical Test  [Compounds] p-value Correction | Update peak rating, area contribution = 3, CV contribution= 10, FWHM to Base contribution = 5, Jaggedness Contribution = 5, Modality Contribution = 5, Zig-Zag Index Contribution = 5  Peak Area  log-10 areas for p-value estimation  The p-value of per group ratio calculated by a two-tailed student's t-test  p-value adjusted using Benjamini-Hochberg correction for the false-discovery rate |

^a^The differential analysis is only employed when spiked TLE-samples is compared to unspiked ones. During each comparison, three technical replicates per spiking level are grouped into their corresponding categorical group. The ratios generated by the hypothesis test have the unspiked group as denominator (e.g. level 4/unspiked TLE).

# Results

## Supplemental table S5: Mean values of retention time (RT) and peak areas of 14 eicosanoid standards included in the SST mix (50 ng/mL each + 50 µg/mL BHT in 1:1 H₂O:MeOH, v/v), measured in full-scan (MS^1^) mode over 21 days (triplicate injections per day).

| **Analyte** | **RT** | **Peak Area** |
| --- | --- | --- |
|  | mean / min | mean / a.u. |
| Tetranor-PGDM | 3.89 | 1.57E+08 |
| 20-hydroxy-PGE2 | 6.86 | 9.58E+07 |
| 6-keto-PGF1a | 9.60 | 2.34E+08 |
| PGF3a | 10.89 | 3.93E+08 |
| PGD2 | 10.89 | 3.93E+08 |
| 8-iso-15-keto- PGE2 | 11.85 | 3.13E+09 |
| PGF2a | 12.16 | 3.68E+08 |
| LTC4 | 15.24 | 9.31E+07 |
| 5(S),15(S)-DiHETE | 15.57 | 2.79E+08 |
| 12(S)-HHT | 16.97 | 8.01E+07 |
| 12(S)-HEPE | 18.42 | 2.63E+08 |
| 15(S)-HETE | 19.27 | 3.17E+08 |
| 12-oxo-ETE | 19.72 | 1.91E+08 |
| (+/-)8,9-EpETrE | 21.00 | 2.49E+07 |


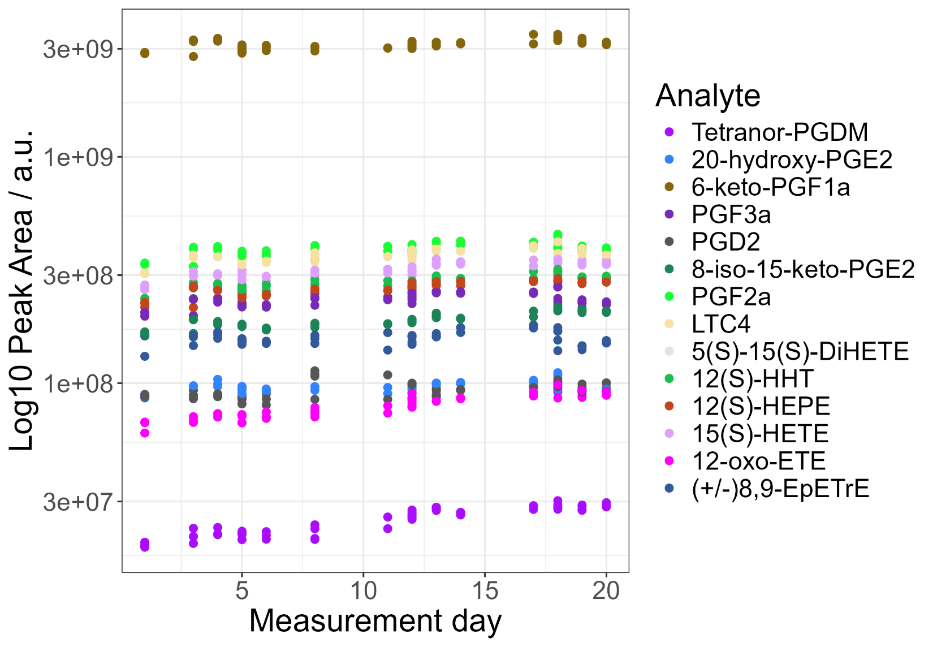


## Supplemental figure S4- Peak areas of 14 eicosanoid standards measured in StdMix samples over 21 consecutive measurement days. The standards were present at 50 ng/mL each, with 50 ng/mL BHT in 1:1 H₂O:MeOH (v/v), and analyzed in triplicate each day. Measurements were performed in full-scan mode and under the same chromatographic conditions described in Figure 1. Peak area variability remained within a coefficient of variation (CV) range of 5–14% across the monitored period, demonstrating stable ionization efficiency and consistent signal response under the applied LC-MS conditions.

## Supplemental table S6- Intensity variation of three spiked eicosanoid standards in TLE (10 ng/mL; Tetranor-PGDM, 6-keto-PGF₁α, and LTC₄) across three weekly measurements using AcquireX. Each standard represents a distinct retention time region of the gradient: early (Tetranor-PGDM), middle (6-keto-PGF₁α), and late (LTC₄).

|  | Week 1 | Week 2 | Week 3 |
| --- | --- | --- | --- |
| Tetranor-PGDM | 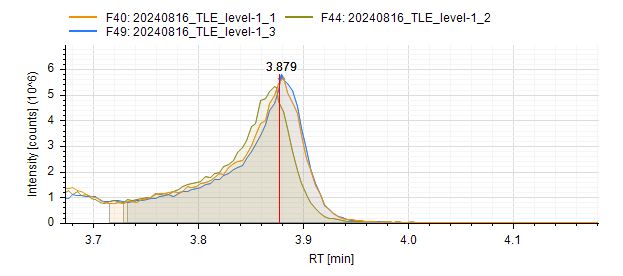 | 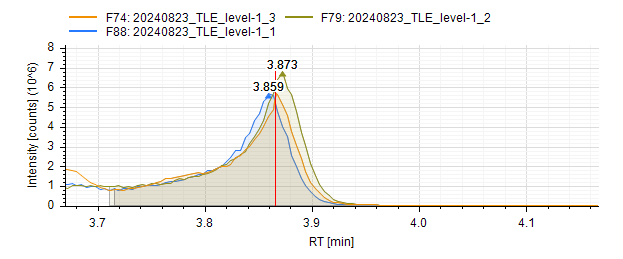 | 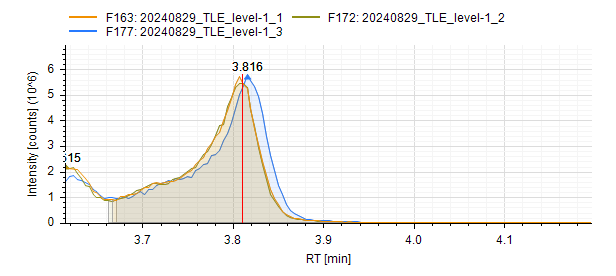 |
| 6-keto-PGF1a | 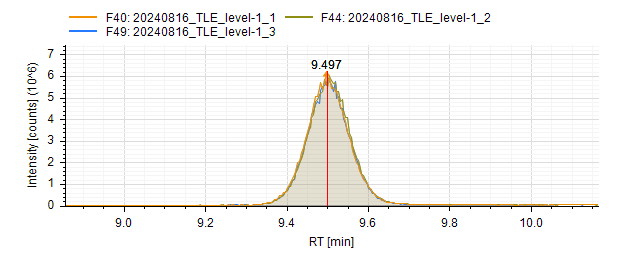 | 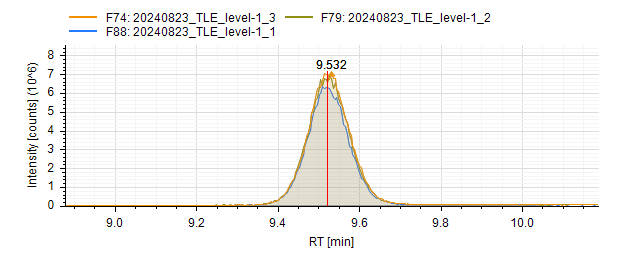 | 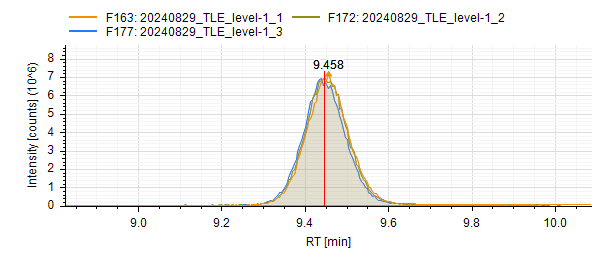 |
| LTC4 | 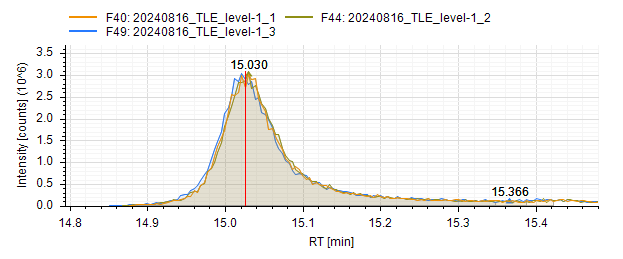 | 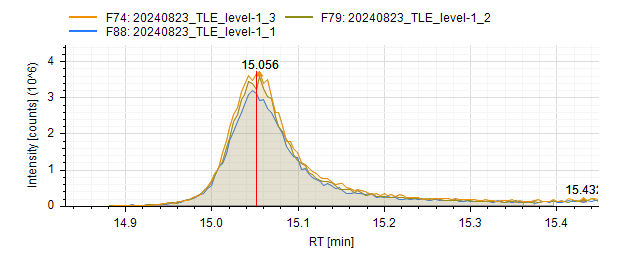 | 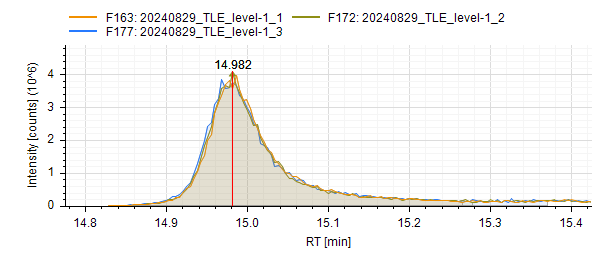 |

## Supplemental table S7- Intensity variation of three spiked eicosanoid standards in TLE (10 ng/mL; Tetranor-PGDM, 6-keto-PGF₁α, and LTC₄) across three weekly measurements using DDA. Each standard represents a distinct retention time region of the gradient: early (Tetranor-PGDM), middle (6-keto-PGF₁α), and late (LTC₄).

|  | Week 1 | Week 2 | Week 3 |
| --- | --- | --- | --- |
| Tetranor-PGDM | 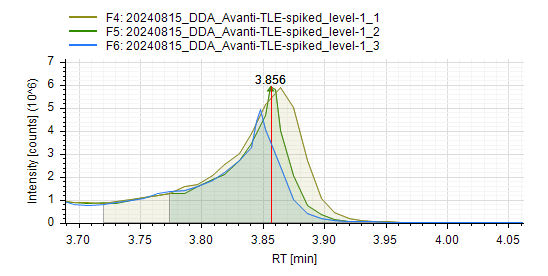 | 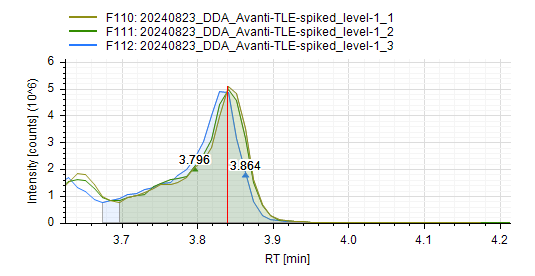 | 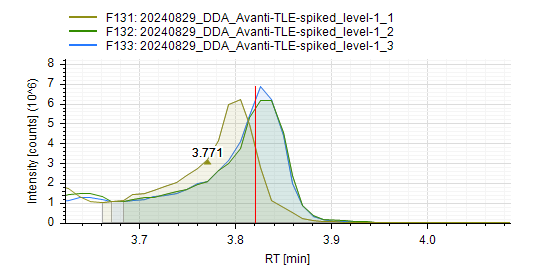 |
| 6-keto-PGF1a | 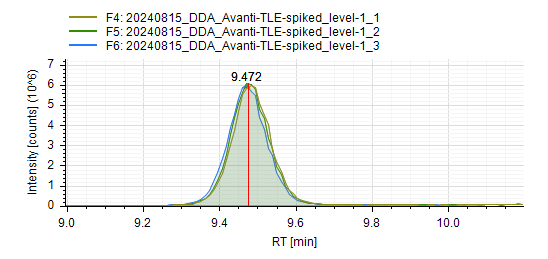 | 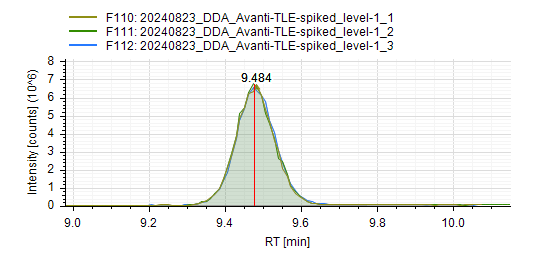 | 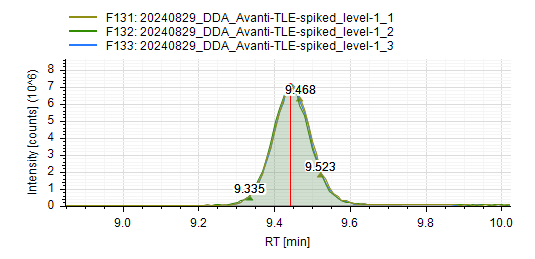 |
| LTC4 | 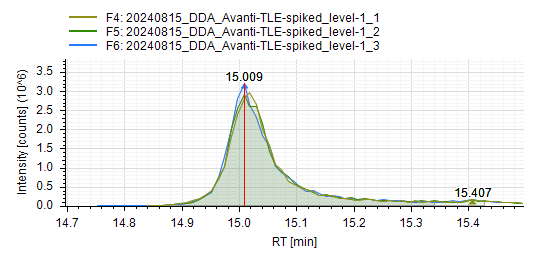 | 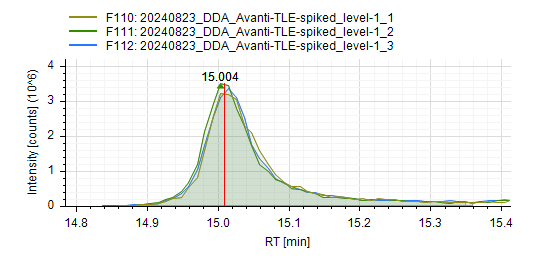 | 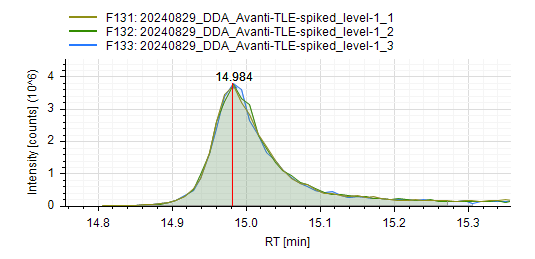 |

## Supplemental table S8- Intensity variation of three spiked eicosanoid standards in TLE (10 ng/mL; Tetranor-PGDM, 6-keto-PGF₁α, and LTC₄) across three weekly measurements using DIA. Each standard represents a distinct retention time region of the gradient: early (Tetranor-PGDM), middle (6-keto-PGF₁α), and late (LTC₄).

|  | Week 1 | Week 2 | Week 3 |
| --- | --- | --- | --- |
| Tetranor-PGDM | 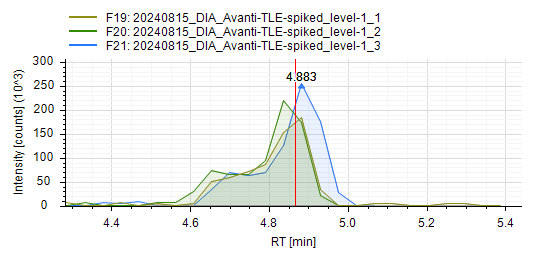 | 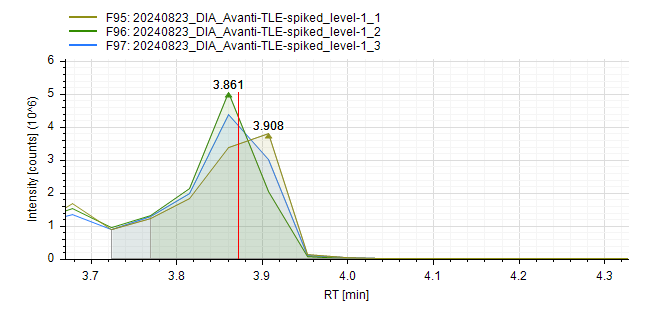 | 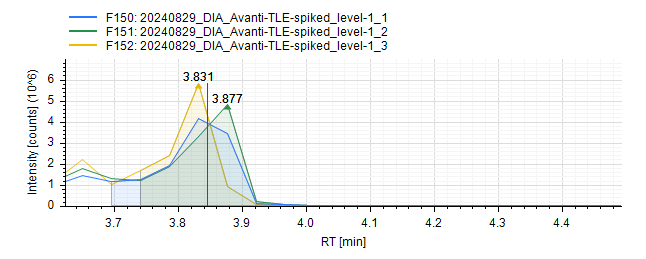 |
| 6-keto-PGF1a | 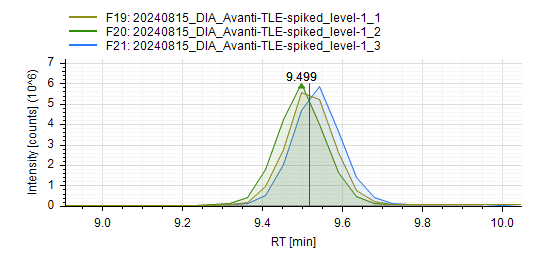 | 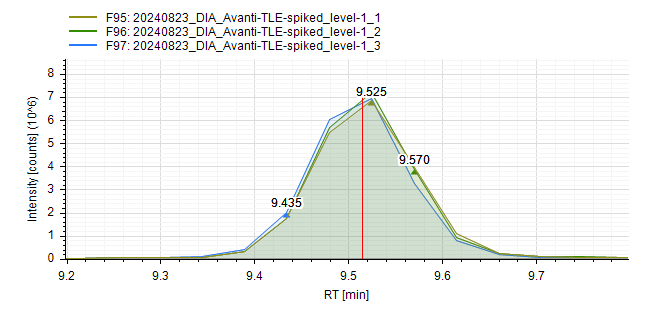 | 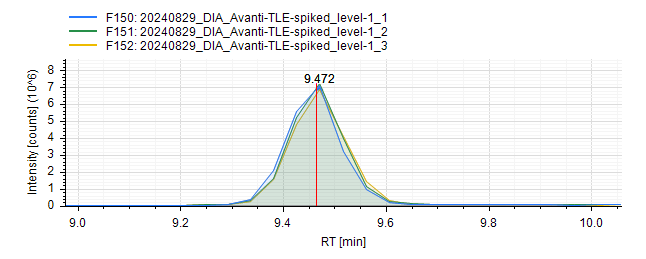 |
| LTC4 | 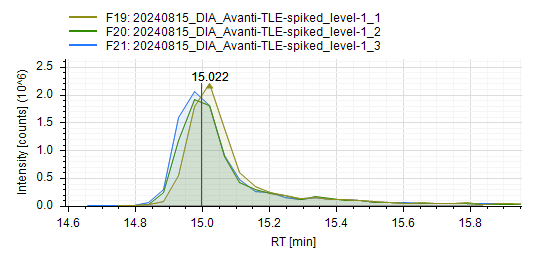 | 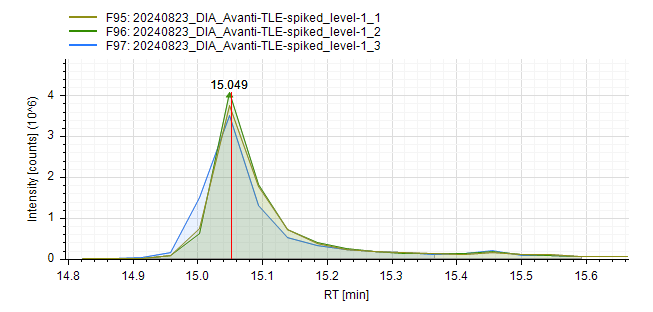 | 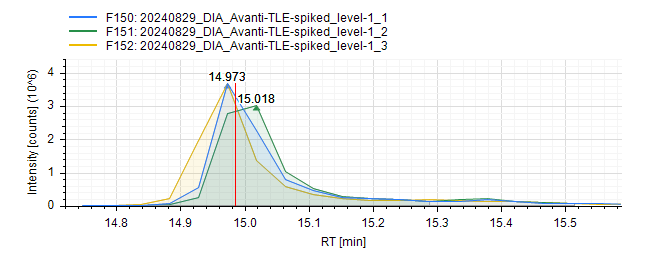 |

## Supplemental table S9- Fragmentation spectra generated for 12(S)-HEPE ([M-H]^-1^ with theoretical m/z 317.2122) with DDA and DIA across weekly measurements at spiking level 10 ng/mL.

|  | **DDA** | **DIA** |
| --- | --- | --- |
| Week 1 | 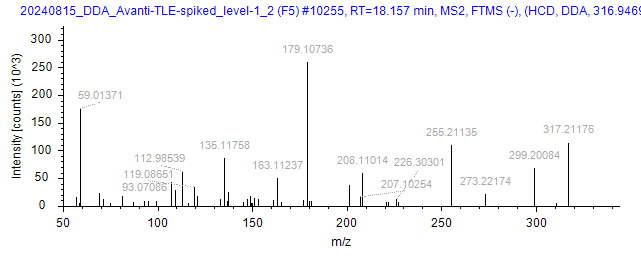  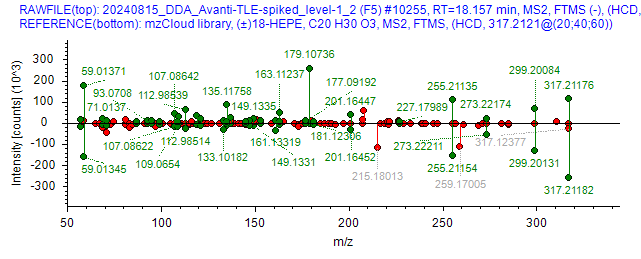  Match score with (+)-18-HEPE of 67.6% and confidence of 55.9% | 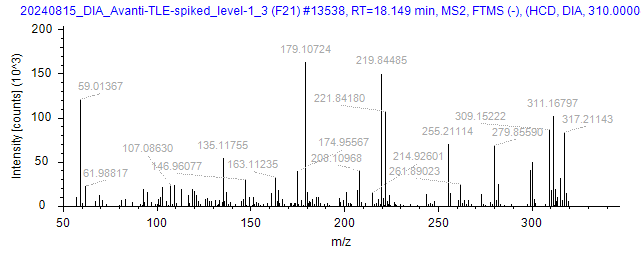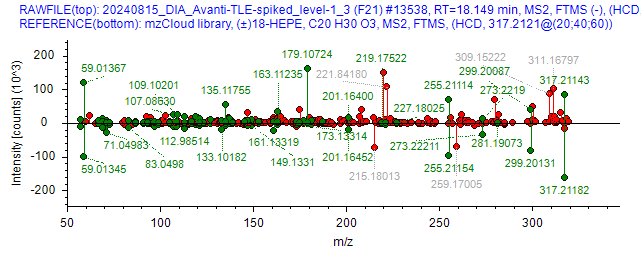  Match score with (+)-18-HEPE of 41.6% and confidence of 49.7% |
| Week 2 | 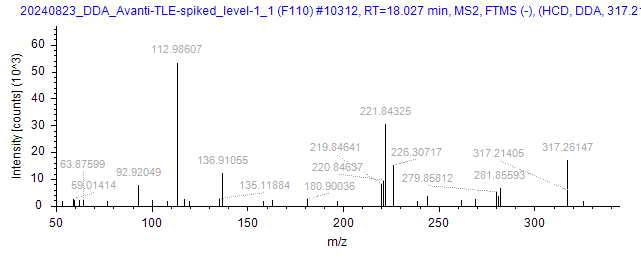  No match | 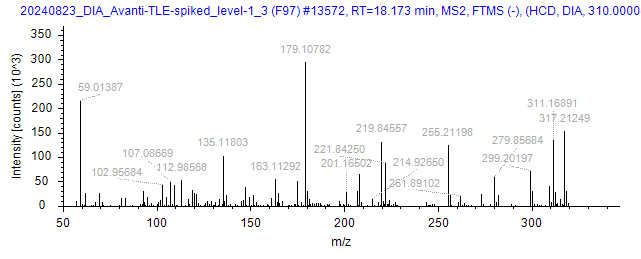  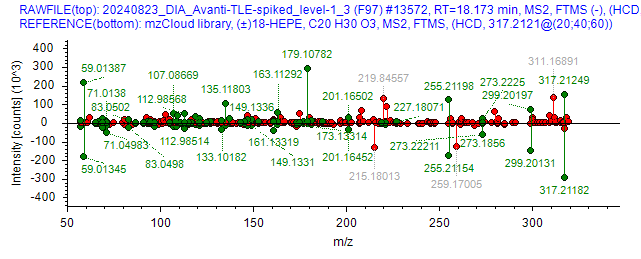  Match score with (+)-18-HEPE of 50.4% and confidence of 51.8% |
| Week 3 | 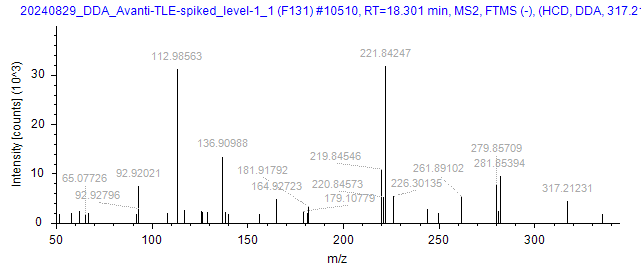  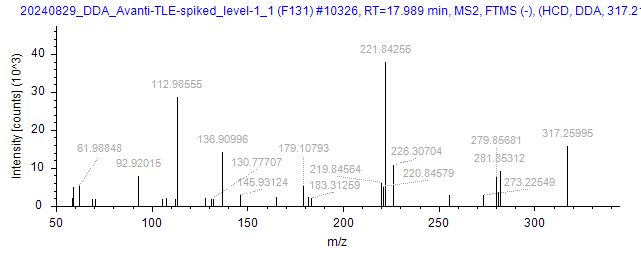  2 MS^2^ spectra were generated by DDA, no match | 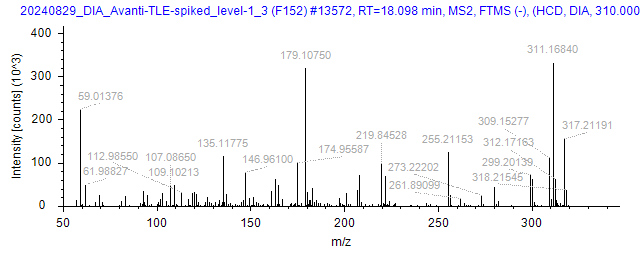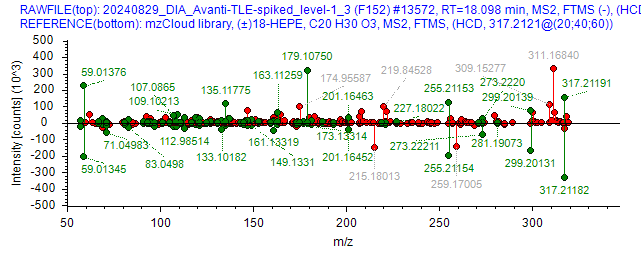Match score with (+)-18-HEPE of 44.3% and confidence of 50.3% |

## Supplemental table S10- Fragmentation spectra generated for LTC4 ([M-H]^-1^ with theoretical m/z 624.296) with DDA and DIA across weekly measurements at spiking level 10 ng/mL (with molecular ion detail).

|  | **DDA** | **DIA** |
| --- | --- | --- |
| Week 1 | 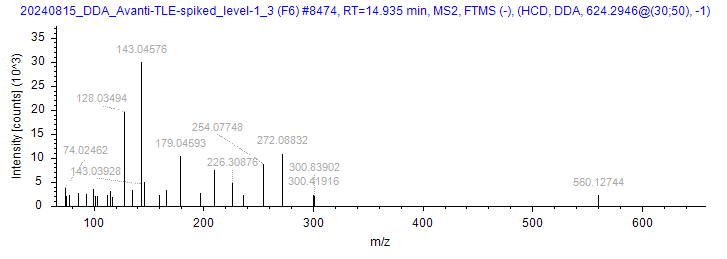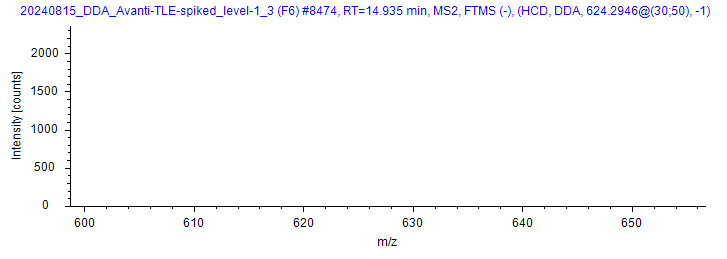  No molecular ion-> no MS^2^ matching. | 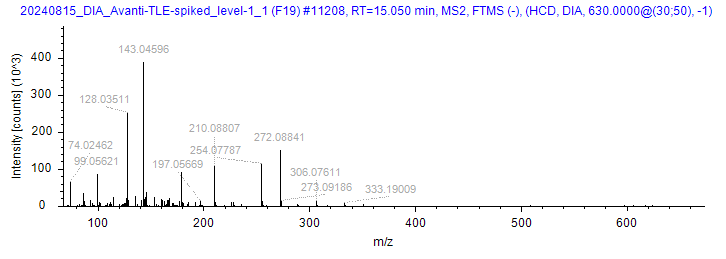  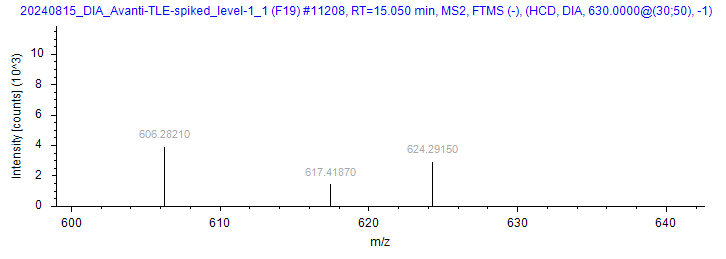  Matched to 11-trans LTC4 with score 66.8% and confidence 47% |
| Week 2 | 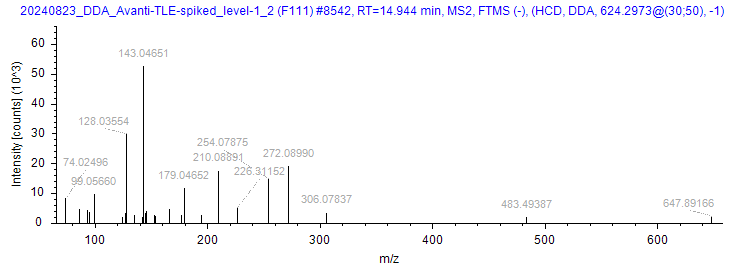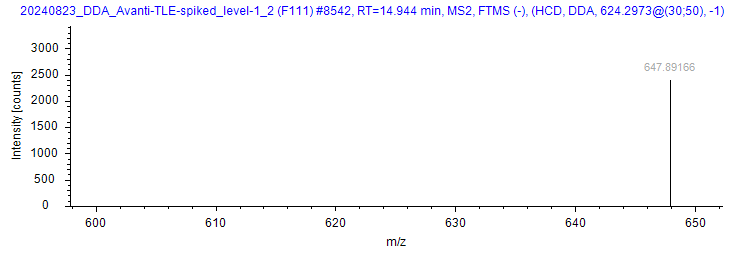  No molecular ions -> no MS^2^ matching | 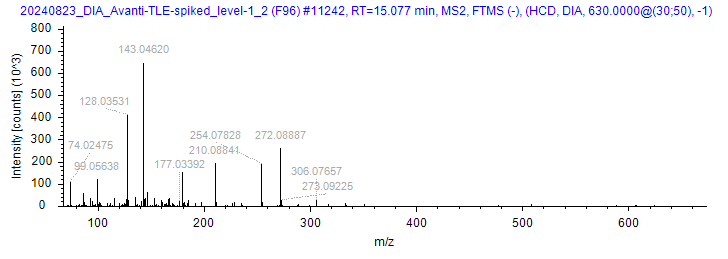  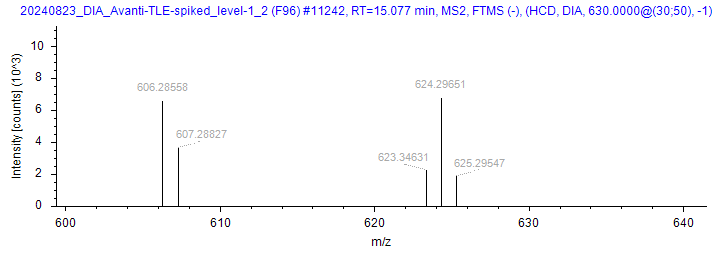  Matched to 11-trans LTC4 with score 68.4% and confidence 47.4% |
| Week 3 | 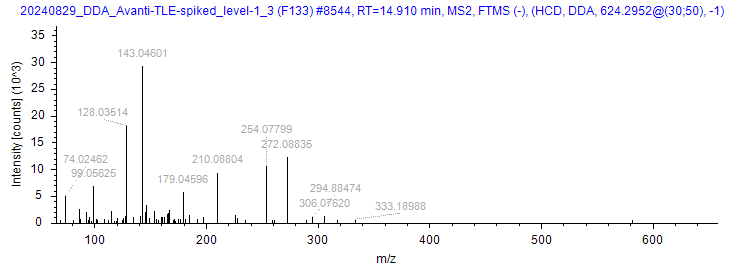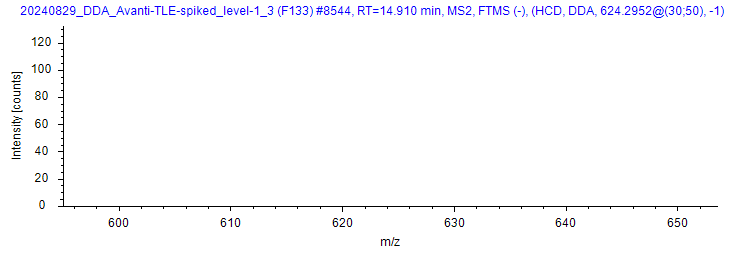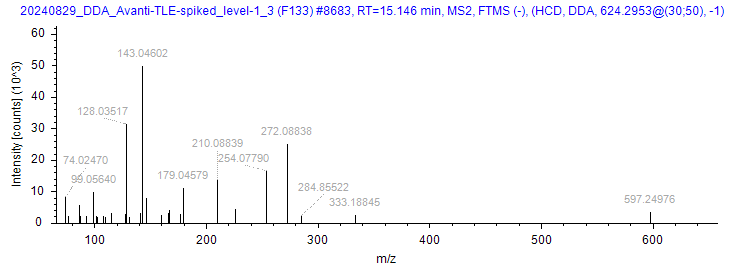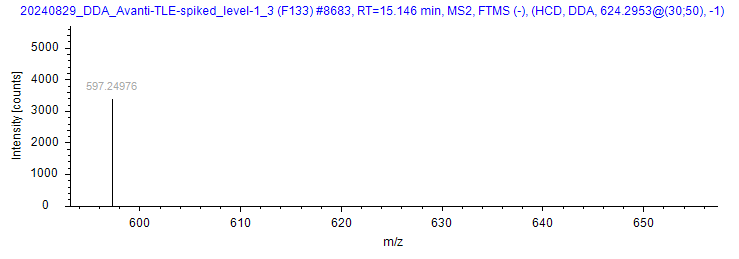  No molecular ion -> no MS^2^ matching | 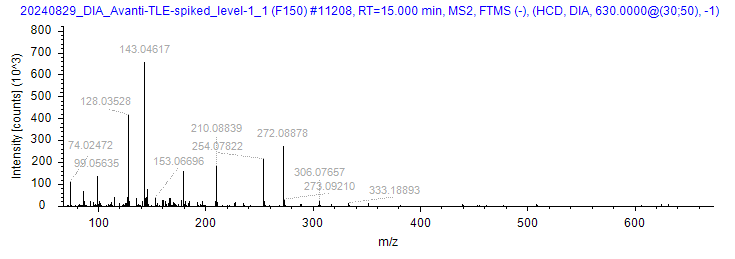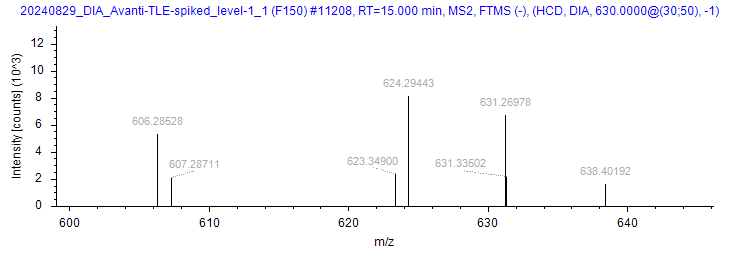  Matched to 11-trans LTC4 with score 68% and confidence 47.3% |

## Supplemental table S11- Fragmentation spectra generated for 6-keto-PGF1α ([M-H]^-1^ with theoretical m/z 369.2283) with DDA and DIA across weekly measurements at spiking level 10 ng/mL.

|  | **DDA** | **DIA** |
| --- | --- | --- |
| Week 1 | 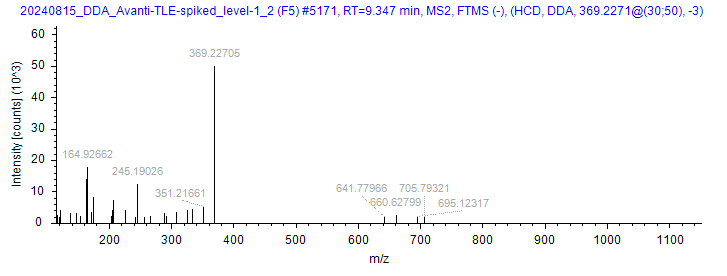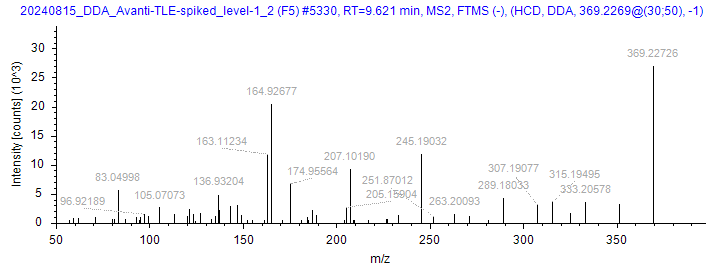  Top spectrum was matched-> correct identification! Match score 75.5% and confidence 63% | 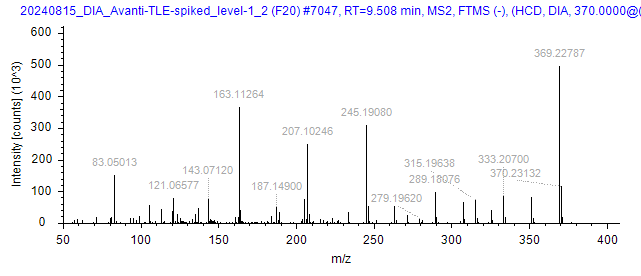 |
| Week 2 | 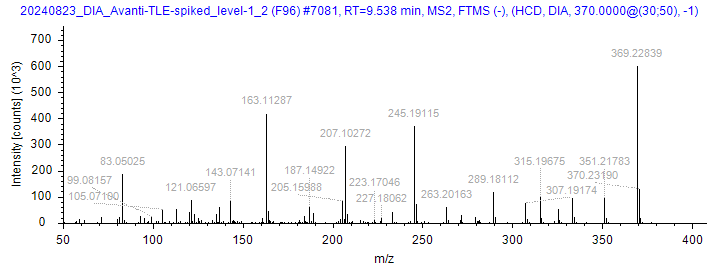  Matching to another isomer with score 78.3% and confidence 66.2% | 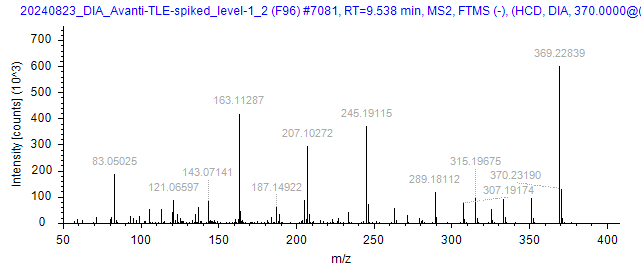 |
| Week 3 | 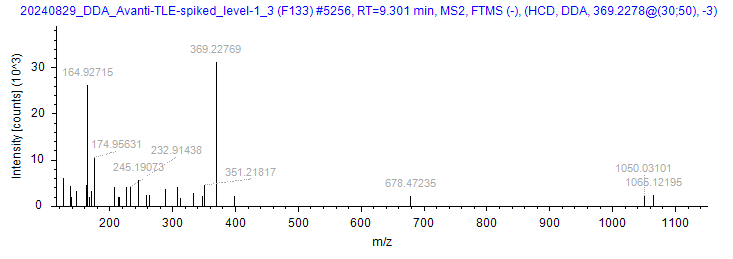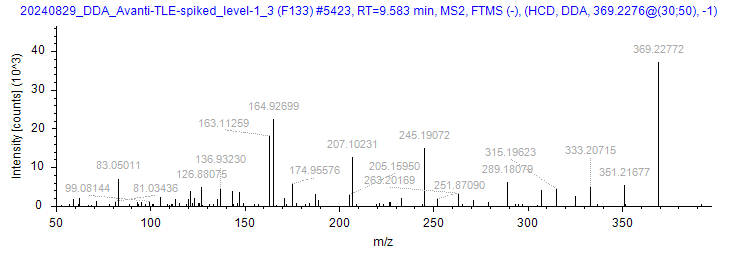  Bottom spectrum was matched- wrong identification!  The correct standard was also matched this time but with lower score and confidence! | 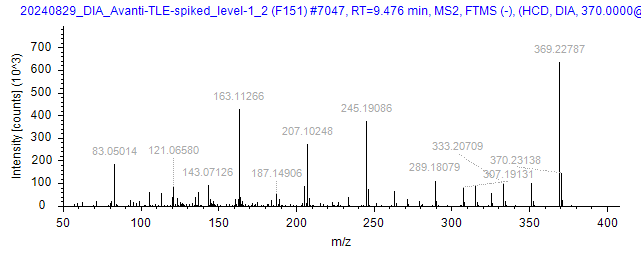 |

## Supplemental table S12- Fragmentation spectra generated for 8-iso-15-keto-PGE2 ([M-H]^-1^ with theoretical m/z 349.202) with AcquireX and DIA across weekly measurements at spiking level 10 ng/mL. The spiking level of 0.01 ng/mL was used for MS^2^ spectral acquisition with AcquireX.

|  | **AcquireX** | **DIA** |
| --- | --- | --- |
| Week 1 | No MS^2^ matching | Correct identification: matching to 8-iso-15-keto-PGE2 with score 94.4% and confidence 86.8% |
| Week 2 | Matching to 19(R)-hydroxy PGE2 with sore 66.5% and confidence 55.6% | Correct identification: matching to 8-iso-15-keto-PGE2 with score 94% and confidence 86.3% |
| Week 3 | No MS^2^ matching. | Correct identification: matching to 8-iso-15-keto-PGE2 with score 94.5% and confidence 87.1% |

## Supplemental table S13- Fragmentation spectra generated for 5(S)-15(S) DiHETE ([M-H]^-1^ with theoretical m/z 335.2228) with AcquireX, DDA, and DIA across weekly measurements at spiking level 10 ng/mL. Spiking level of 0.01 ng/mL was used for MS^2^ spectral acquisition with AcquireX.

|  | **AcquireX** | **DDA** | **DIA** |
| --- | --- | --- | --- |
| Week 1 | No MS^2^ spectrum acquired. | Correct identification: matching to 5(S)-15(S) DiHETE with score 86.3% and confidence 75.6% | Correct identification: matching to 5(S)-15(S) DiHETE with score 89.2% and confidence 79% |
| Week 2 | Matching to another isomer with score 73.5% and confidence 60.6% | Correct identification: matching to 5(S)-15(S) DiHETE with score 75.4% and confidence 62.8% | Correct identification: matching to 5(S)-15(S) DiHETE with score 90.2% and confidence 80.3% |
| Week 3 | No MS^2^ spectrum acquired. | Correct identification: matching to 8-iso-15-keto-PGE2 with score 82.6% and confidence 71.2% | Correct identification: matching to 5(S)-15(S) DiHETE with score 89.1% and confidence 78.8% |

## Supplemental table S14- Fragmentation spectra generated for coeluting PGF3α and PGD2 ([M-H]^-1^ with theoretical m/z 351.2177) with DDA and DIA across three weekly measurements. Orange circles highlight fragment ions consistent with PGF₃α (m/z 307, 245, 193, and 171), and green circles indicate fragments characteristic of PGD₂ (m/z 315, 271, and 233). The mixed fragmentation pattern reflects the coelution and differing ionization efficiencies of these isomers.

|  | **DDA** | **DIA** |
| --- | --- | --- |
| Week 1 | Top one used for MS^2^ matching. Matching with PGF3α with score 75% and confidence 62.4%. | Matching with PGF3α with score 85.7% and confidence 74.9%. The 2^nd^ image is only a close-up of the 1^st^ one. |
| Week 2 | No match, wrong annotation. | Matching with PGF3α with score 87.2% and confidence 76.7%. The 2^nd^ image is only a close-up of the 1^st^ one. |
| Week 3 | Top one used for MS^2^ matching. Matching with PGF3α with score 81.5% and confidence 70%. | Matching with PGF3α with score 86.8% and confidence 76.2%. The 2^nd^ image is only a close-up of the 1^st^ one. |

## Supplemental table S15- MS¹ spectra for PGF₂α ([M-H]^-1^ with theoretical m/z 353.2333) at 0.01 ng/mL spiked level acquired by DDA and DIA (week 3).

| **DDA** | **DIA** |
| --- | --- |
|  |  |
